# Multifunctional Sr/Se co-doped ZIF-8 nanozyme for chemo/chemodynamic synergistic tumor therapy *via* apoptosis and ferroptosis

**DOI:** 10.7150/thno.92663

**Published:** 2024-02-24

**Authors:** Aimin Wu, Ming Han, Zihan Ni, Haoran Li, Yinyin Chen, Zhouping Yang, Yumei Feng, Zufeng He, Hua Zhen, Xianxiang Wang

**Affiliations:** 1 Institute of Animal Nutrition, Sichuan Agricultural University, Chengdu 611130, Sichuan, China.; 2 College of Science, Sichuan Agricultural University, Chengdu 611130, Sichuan, China.; 3 College of Agronomy, Sichuan Agricultural University, Chengdu 611130, Sichuan, China.; 4 State Key Laboratory of Crop Gene Exploration and Utilization in Southwest China, Chengdu 611130, Sichuan, China.; 5 Institute of New Rural Development, Sichuan Agricultural University, Chengdu 611130, Sichuan, China.; 6 College Veterinary Medicine, Sichuan Agricultural University, Chengdu 611130, Sichuan, China.

**Keywords:** Apoptosis, Doxorubicin, Ferroptosis, Tumor therapy, ZIF-8/SrSe

## Abstract

**Rationale:** Cancer continues to be a significant public health issue. Traditional treatments such as surgery, radiotherapy, and chemotherapy often fall short because of intrinsic issues such as lack of specificity and poor drug delivery, leading to insufficient drug concentration at the tumor site and/or potential side effects. Consequently, improving the delivery of conventional chemotherapy drugs like doxorubicin (DOX) is crucial for their therapeutic efficacy. Successful cancer treatment is achieved when regulated cell death (RCD) of cancer cells, which includes apoptotic and non-apoptotic processes such as ferroptosis, is fundamental to successful cancer treatment. The developing field of nanozymes holds considerable promise for innovative cancer treatment approaches.

**Methods:** A dual-metallic nanozyme system encapsulated with DOX was created, derived from metal-organic frameworks (MOFs), designed to combat tumors by depleting glutathione (GSH) and concurrently liberating DOX. The initial phase of the study examined the GSH oxidase-mimicking function of the dimetallic nanozyme (ZIF-8/SrSe) through enzyme kinetic assays and Density Functional Theory (DFT) simulations. Following this, we probed the ability of ZIF-8/SrSe@DOX to release DOX in response to the tumor microenvironment *in vitro*, alongside examining its anticancer capabilities and mechanisms prompting apoptosis or ferroptosis in cancer cells. Moreover, we established tumor-bearing animal models to corroborate the anti-tumor effectiveness of our nanozyme complex and to identify the involved apoptotic and ferroptotic pathways implicated.

**Results:** Enzyme kinetic analyses demonstrated that the ZIF-8/SrSe nanozyme exhibits substantial GSH oxidase-like activity, effectively oxidizing reduced GSH to glutathione disulfide (GSSG), while also inhibiting glutathione peroxidase 4 (GPX4) and solute carrier family 7 member 11 (SLC7A11). This inhibition led to an imbalance in iron homeostasis, pronounced caspase activation, and subsequent induction of apoptosis and ferroptosis in tumor cells. Additionally, the ZIF-8/SrSe@DOX nanoparticles efficiently delivered DOX, causing DNA damage and further promoting apoptotic and ferroptotic pathways.

**Conclusions:** This research outlines the design of a novel platform that combines chemotherapeutic agents with a Fenton reaction catalyst, offering a promising strategy for cancer therapy that leverages the synergistic effects of apoptosis and ferroptosis.

## Introduction

Cancer remains a leading cause of global mortality, with a significant impact on public health 1. Currently, the most widely used treatments can be divided into surgery, chemotherapy, and radiation. Chemotherapy, effective in eliminating tumor cells, often causes a range of adverse effects, from minor to severe. Typically, the therapeutic efficacy of doxorubicin (DOX), a commonly used chemotherapeutic agent, is hindered by its poor tumor specificity and short plasma half-life, leading to low drug concentration at the tumor site and non-selective toxicity not only affects malignant cells but also impairs healthy cells; this necessitates higher dosages for effective treatment, increasing the risk of systemic toxicity and potentially leading to tumor cell chemoresistance, thereby risking treatment failure 2. The urgent need for more tumor-specific drug delivery systems for DOX is clear. Nanozymes are nanomaterials that display intrinsic enzyme-like properties 3, such as metal oxides, sulfides, monoatoms and metal-organic frameworks. They are considered attractive for diagnostic drugs, biosensing and targeted therapies due to their excellent properties such as biocompatibility, stability, low cost, good targeting, controlled drug release and other excellent properties 4-6. The joint application of multifunctional nanoplatforms with tumor microenvironment (TME)-responsive specific cancer therapy to construct TME-responsive smart nanoplatforms has attracted more and more attention in the field of nanomedicine. These nanoplatforms have been confirmed to have great advantages and better efficacy than traditional tumor treatments 7. Among them, zeolite imidazolate frameworks (ZIFs) are promising as carriers, thanks to their large pore volumes and high surface areas, which improve the delivery of therapeutic agents to targeted tissues 8. Moreover, these nanoporous structures are uniquely sensitive to the TME, reacting to elevated temperatures (above 37°C) and acidic pH levels, making them strong candidates for innovative drug delivery systems.

The porous nature of ZIF-8 is frequently leveraged for drug delivery due to its instability in acidic conditions, and the addition of different metal ions can enhance its properties 9. Selenium (Se), vital for human health, acts as the active center of several enzymes and has key roles in disease prevention, anti-aging, immune function, and cancer protection 10-12. However, research indicates that excessive Se intake can upset redox balance, causing protein thiol group oxidation, cross-linking, and increased production of reactive oxygen species (ROS), leading to notable anticancer effects, thus piquing interest in its use in cancer pharmacotherapy 13. The narrow therapeutic index of Se marks a thin line between its advantageous and harmful doses, limiting its clinical application in cancer treatment. In contrast, strontium (Sr), another crucial trace element, has been recognized for its diverse biological functions 14, including strengthening bone and dental health 15, influencing reproductive health 16, and exerting antitumor effects both in living organisms and in laboratory settings. Bai et al. 17 showed that Sr could reduce chromosomal damage induced by polycyclic aromatic hydrocarbons (PAHs), indicating its potential as a new anticancer compound. Despite these findings, research on Sr's anticancer applications is still in the nascent stages.

Regulated cell death (RCD) is a crucial strategy in cancer therapy. RCD is governed by signal transduction pathways with clearly delineated mechanisms of action. This biological process can be primarily classified into apoptotic or non-apoptotic categories, each with unique morphological, biochemical, immunological, and genetic traits 18, 19. Apoptosis, a well-documented variant of programmed cell death, typically proceeds via caspase-dependent processes triggered by diverse factors like chemotherapeutic agents, hyperthermia, and ROS 20, 21. Tumor cells exhibit the capacity for uncontrolled multiplication, leading to atypical growth and vigorous cellular respiration that heightens ROS levels. To counterbalance this, glutathione (GSH), a prevalent intracellular reductant, is amplified to detoxify these ROS and inhibit oxidative imbalances 22. However, cancer cells increasingly adapt to ROS-based interventions, cultivating resistance to apoptotic triggers and diminishing the effectiveness of standalone anti-cancer therapies 23. Consequently, research has pivoted towards additional RCD pathways, notably ferroptosis, autophagy, and necroptosis, to bypass such resistance. Ferroptosis represents a promising form of programmed necrotic cell death, particularly in combating resistance to conventional apoptosis and enhancing the eradication of tumor cells. Central to ferroptosis is the accumulation of ferric ions that, when reacting with hydrogen peroxide via the Fenton reaction, yield potent hydroxyl radicals (·OH), propelling the process of lipid peroxidation in tumor cells 24, 25. This is further compounded by the downregulation of antioxidant systems such as GSH and glutathione peroxidase 4 (GPX4), culminating in impaired ROS detoxification and fostering an environment conducive to lipid peroxidation and ferroptosis 26, 27. Therefore, a combined therapeutic approach integrating apoptosis with ferroptosis offers a comprehensive and potentially more effective cancer treatment strategy 28, 29.

**Scheme 1:**
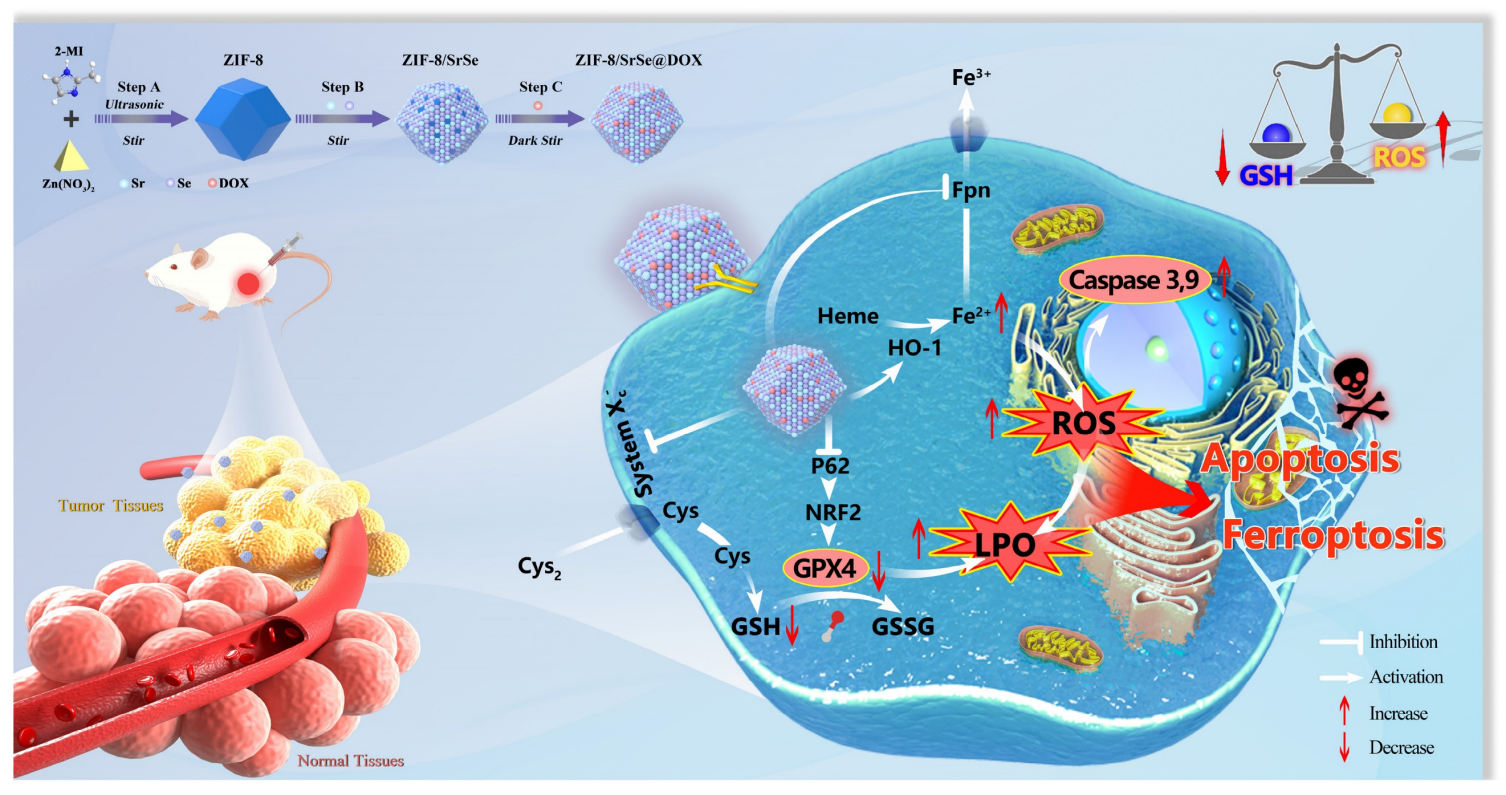
ZIF-8/SrSe@DOX nanoparticles exhibiting exceptional GSH oxidase-like activity for synergistic apoptosis-ferroptosis tumor therapy.

In this study, we have successfully developed a novel nanozyme construct termed DOX-loaded ZIF-8/SrSe. Our goal was to design and synthesize a platform that merges chemotherapeutic agents with a Fenton reaction catalyst, aiming for a synergistic approach to cancer treatment by simultaneously inducing apoptosis and ferroptosis. This cutting-edge therapeutic strategy combines the mechanisms of chemotherapy and chemodynamic therapy (CDT) to enhance the treatment's efficacy. We synthesized ZIF-8/SrSe using a solvent method and subsequently encapsulated it with DOX, resulting in the innovative ZIF-8/SrSe@DOX complex (Scheme 1). The co-doping of Sr and Se into the ZIF-8 unexpectedly imparted significant glutathione oxidase-mimetic activity. This enhancement led to the oxidation of reduced GSH to its oxidized form GSSG while simultaneously inhibiting both GPX4 and SLC7A11. Moreover, this bifunctional activity modulates iron metabolic pathways by influencing iron uptake and translocation through Transferrin Receptor 1 (TFR1) and Ferroportin (FPN), and impacting iron storage by affecting both the heavy and light-chain subunits of ferritin (FTH/L), as well as significantly upregulating heme oxygenase 1 (HO-1). These interferences in the SLC7A11-GSH-GPX4 signaling axis, paired with imbalances in iron homeostasis, were shown to greatly heighten ferroptosis in tumor cells, as corroborated by both *in vitro* and *in vivo* evidence. When introduced, ZIF-8/SrSe@DOX markedly diminishes the expression of nuclear factor erythroid 2-related factor 2 (NRF2) and its associated protein p62, which facilitates the progression of ferroptosis in tumor cells. Further examination showed that ZIF-8/SrSe@DOX treatment pronouncedly triggers caspase activation, culminating in the apoptosis of tumor cells. Within the TME, ZIF-8/SrSe@DOX responds to increased levels of GSH and acidic conditions, prompting its decomposition, allowing for the controlled release of the chemotherapeutic agent DOX. This release not only elicits DNA damage, ensuing in apoptosis, but also partly induces ferroptosis.

## Results and Discussion

### Preparation and characterization of ZIF-8/SrSe@DOX

**Figure 1:**
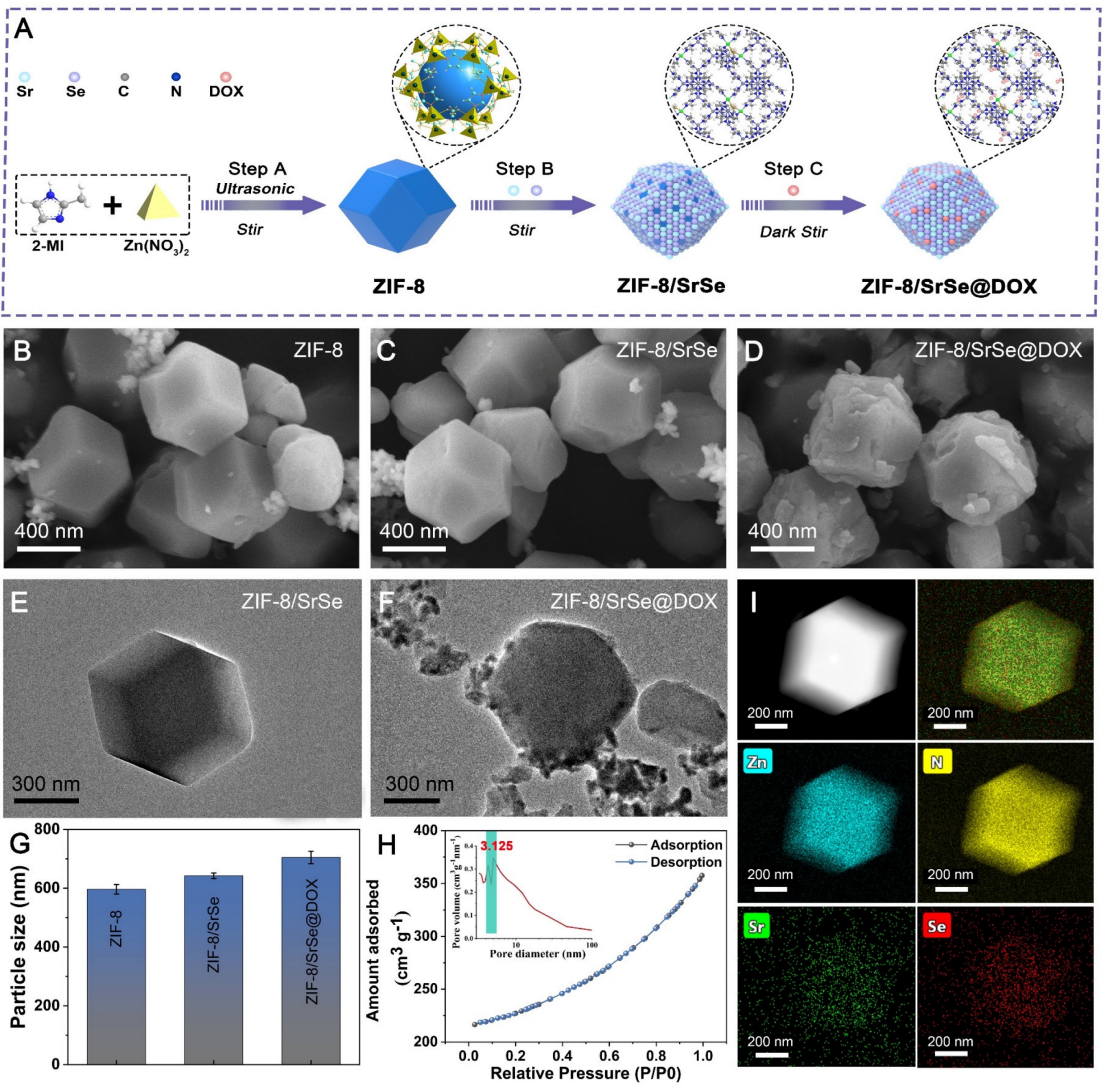
** Characterization of nanomaterials.** (A) Schematic representation of the synthesis process for ZIF-8/SrSe@DOX NPs. (B) SEM image of ZIF-8, C) ZIF-8/SrSe and D) ZIF-8/SrSe@DOX NPs. (E) HRTEM image of ZIF-8/SrSe and F) ZIF-8/SrSe@DOX. (G) Comparison of size distribution among ZIF-8, ZIF-8/SrSe, and ZIF-8/SrSe@DOX. (H) Nitrogen adsorption-desorption isotherm, pore size distribution plot and I) TEM mapping of the ZIF-8/SrSe.

The synthesis procedure of ZIF-8/SrSe@DOX is depicted in Figure 1A. Initially, nanosized ZIF-8 were synthesized using Zn(NO_3_)_2_ and 2-methyl imidazole as precursors, following a modified method 30. The pre-synthesized ZIF-8 nanoparticles were then vigorously stirred with strontium acetate, sodium selenite, and sodium borohydride (NaBH_4_), culminating in the successful formation of ZIF-8/SrSe. Subsequently, DOX was efficiently loaded onto ZIF-8/SrSe by exploiting its high specific surface area. When compared to the pristine ZIF-8 (Figure 1B), the composites ZIF-8/SrSe (Figure 1C) and ZIF-8/SrSe@DOX (Figure 1D) displayed only minor morphological alterations, with the noteworthy exception of an increase in particle size to 50 nm for ZIF-8/SrSe and to 60 nm for ZIF-8/SrSe@DOX, as evidenced in (Figure 1G, Figure S1). The particle size increment observed is indicative of the successful entrapment of DOX within the ZIF-8/SrSe structure. TEM images (Figure 1E and F) confirmed that the synthesized nanomaterials retained a consistent hexagonal shape. Brunauer-Emmett-Teller (BET) analysis (Figure 1H) revealed that the ZIF-8/SrSe has a surface area of 707.7 m²/g and an average pore diameter of 3.125 nm, properties favoring drug encapsulation. As expected, encapsulation of guest molecules within ZIF-8/SrSe resulted in decreased surface area and pore volume, evidenced by the reduced values of 506.5 m²/g and 2.489 nm, respectively, for ZIF-8/SrSe@DOX (Figure S2, Table S1). This reduction is attributable to the accommodation of DOX molecules within the pores of the framework. Elemental mapping of ZIF-8/SrSe further confirmed the integration of Zn, Sr, and Se elements within the composite (Figure 1I).

**Figure 2:**
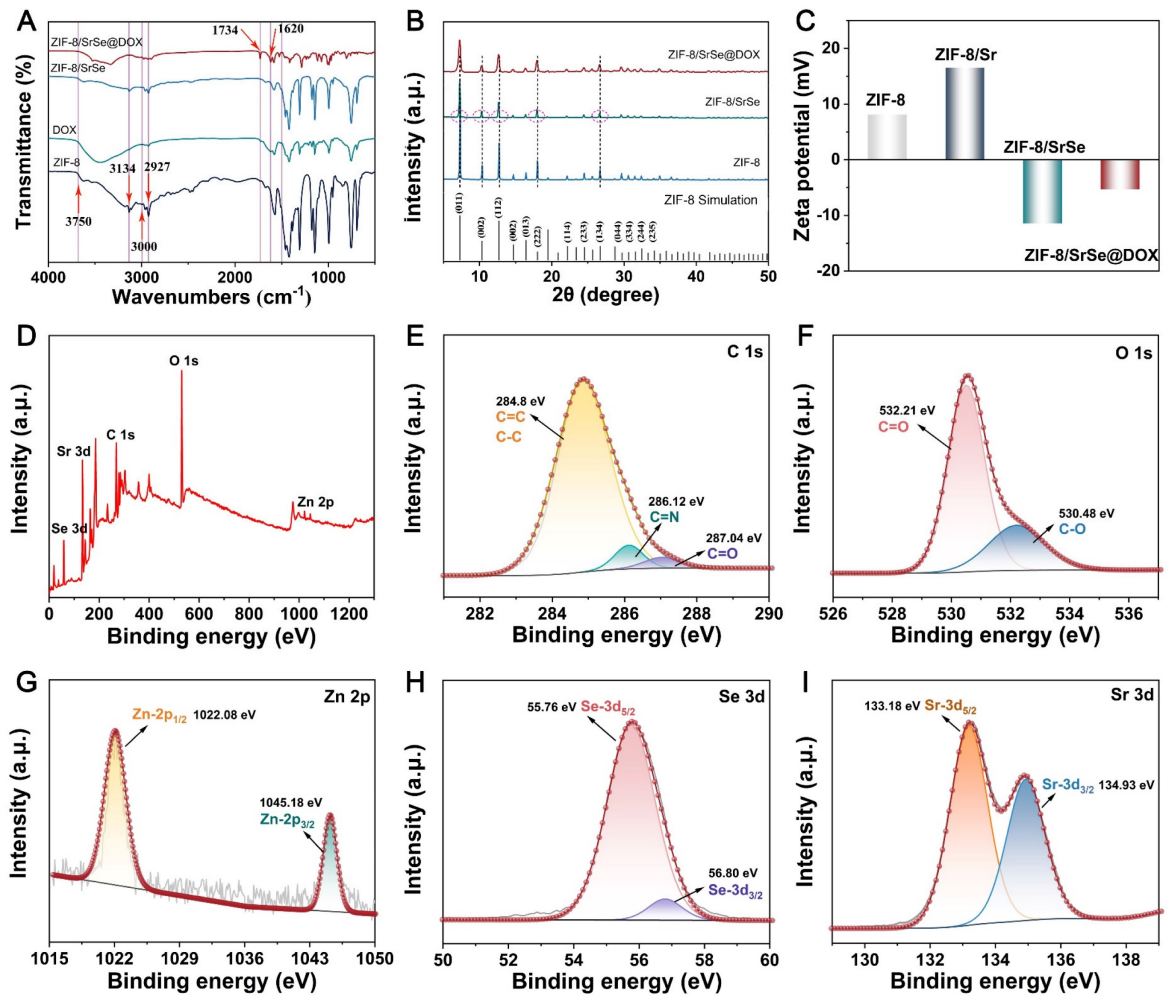
(A) FTIR spectra, B) XRD patterns and C) Zeta potentials of ZIF-8, ZIF-8/SrSe and ZIF-8/SrSe@DOX. (D) XPS survey spectra of ZIF-8/SrSe. (E) C 1s, F) O 1s, G) Zn 2p, H) Se 3d and I) Sr 3d XPS spectra of ZIF-8/SrSe.

Fourier-transform infrared (FTIR) spectroscopy was employed to corroborate the successful encapsulation of DOX. The ZIF-8 spectrum displayed distinct absorption bands, with peaks at 2927 and 3134 cm^-1^ consistent with the stretching vibrations of aliphatic and aromatic C-H bonds, respectively. Additionally, the spectrum featured characteristic peaks between 500 and 1500 cm^-1^, which are attributable to the in-plane bending and stretching vibrations of the imidazole ring (Figure 2A) 31. DOX itself manifested characteristic absorption peaks associated with its amino groups at 1620 cm^-1^ and across the 3000-3750 cm^-1^ range, along with a carbonyl group peak at 1734 cm^-1^. These spectral signatures were also present in the FTIR spectrum of ZIF-8/SrSe@DOX, implying the successful integration of DOX into the ZIF-8/SrSe framework. Furthermore, the X-ray diffraction (XRD) patterns of ZIF-8, ZIF-8/SrSe, and ZIF-8/SrSe@DOX corresponded well with the simulated models, indicating that the crystalline structure remained essentially unchanged after DOX loading. Additionally, a minor leftward shift in the diffraction peaks of ZIF-8/SrSe in comparison with those of ZIF-8 provided evidence for the successful doping of Sr and Se ions into the ZIF-8 structure (Figure 2B). Figure 2C demonstrates that undoped ZIF-8 has a zeta potential of +10 mV, as revealed by zeta potential measurements. When doped with Sr, the potential escalates to +19.5 mV, a likely consequence of incorporating Sr^2+^ ions. In stark contrast, the ZIF-8/SrSe composite manifests a zeta potential of -10 mV, indicative of the probable reduction of selenium ions to Se^2-^ within the structure. Furthermore, DOX, which carries a positive charge, upon encapsulation within the ZIF-8/SrSe framework, causes an increase in the zeta potential.

X-ray photoelectron spectroscopy (XPS) analysis was employed to further elucidate the chemical composition and structural features of ZIF-8/SrSe. Calibration of the obtained spectra was performed using the carbon 1s peak at a binding energy of 284.8 eV (Figure 2E). As presented in Figure 2D-I, the survey spectrum comprehensively identifies the elements present in the samples, namely Zn, Sr, Se, C, and O. The spectra of Zn 2p_1/2_ (1022.08 eV) and Zn 2p_3/2_ (1045.18 eV) confirmed the existence of Zn^2+^ (Figure 2G) 32. The peaks observed at 55.76 eV and 56.80 eV can be attributed to the Se 3d_5/2_ and 3d_3/2_ states, respectively, confirming the presence of selenium (Figure 2H). The observation of the Se 3d_5/2_ peak corroborates the incorporation of divalent selenium in its -2 oxidation state, suggesting that selenium ions are capable of adsorption into the ZIF-8 framework 33. The high-resolution XPS spectrum of strontium (Sr 3d) exhibited split peaks at approximately 133.18 eV and 134.93 eV (Figure 2I), corresponding to the Sr 3d_5/2_ and Sr 3d_3/2_ states, respectively 34. The oscillating peak at 133.18 eV suggests the presence of Sr^2+^ ions in the nanozyme. The smaller size of strontium cations (2.26 Å) compared to the window size of ZIF-8 (3.4 Å) allows for the diffusion of Sr^2+^ into the ZIF-8 cages, where it interacts with Im^-^ anions. This interaction between Sr^2+^ and Im^-^ anions is stronger than that between Zn^2+^ and Im^-^, indicating the effective substitution of Zn^2+^ by Sr^2+^ 35. To support this explanation, we performed a calculation of the energy change using the B3LYP/6-31g(d)/SDD method, a hybrid density functional theory (DFT) method, for the proposed substitution reaction.

Zn (Im)_2_+Sr^2+^ = Sr (Im)_2_+Zn^2+^

As shown in (Figure S3, Table S2), bivalent cations (Sr^2+^) can readily substitute Zn^2+^ with a release of energy (-3.459 kcal/mol). This indicates that Sr^2+^ present in a solution can easily replace Zn^2+^ in ZIF-8.

### GSH depletion and cascade catalytic performance

**Figure 3:**
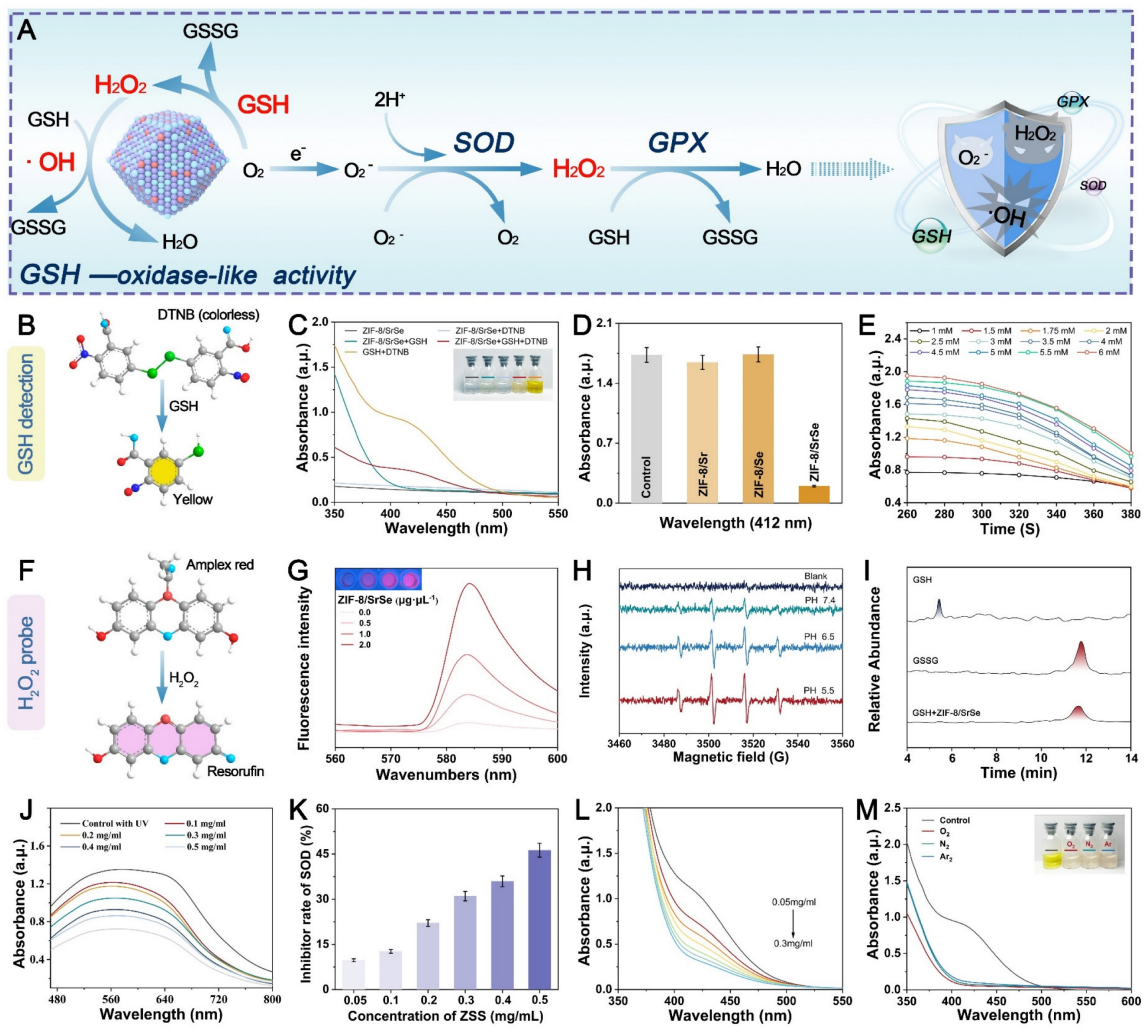
** Relevant enzymatic activities of nanoenzymes.** (A) Schematic representation of the cascade catalytic performance of the NPs. (B) GSH detection using DTNB. (C) Feasibility analysis of GSH consumption by ZIF-8/SrSe at pH 6.5 and 40℃. (D) GSH consumption in different reaction systems: (1) GSH + DTNB, (2) GSH + DTNB + ZIF-8/Sr, (3) GSH + DTNB + ZIF-8/Se, and (4) GSH + DTNB + ZIF-8/SrSe. (E) Time-dependent absorbance upon addition of ZIF-8/SrSe (100 μg·mL^-1^) and varying GSH concentrations (1-6 mM). (F) H_2_O_2_ detection using Amplex Red, resulting in red Resorufin product formation. (G) Generation of H_2_O_2_ after incubation with ZIF-8/SrSe (0.5-2.0 μg·μL^-1^) for 6 hours, as determined by Amplex Red reagent. (H) ESR spectra of •OH trapped by DMPO upon ZIF-8/SrSe. (I) UPLC for GSH, GSSG, and catalytic products (ZIF-8/SrSe + GSH). (J) Catalytic conversion of O_2_·^-^ by different concentrations of ZIF-8/SrSe. (K) SOD-like activity of ZIF-8/SrSe. (L) Reduction of ZIF-8/SrSe in the presence of H_2_O_2_ at different concentrations, leading to GSH depletion. (M) Direct oxidation of ABTS by ZIF-8/SrSe in N_2_, Air, and O_2_ atmosphere.

In comparison to normal cells, tumor cells maintain a redox balance through high concentrations of GSH, which promotes cancer cell proliferation and poses challenges in cancer treatment. To investigate the potential Glutathione Oxidase-like (GSHOx-like) activity of ZIF-8/SrSe, we conducted a 5,5'-dithiobis (2-nitrobenzoic acid) (DTNB) assay 36. Our findings in Figure 3A revealed that ZIF-8/SrSe exhibited significant GSH scavenging activity. The ability of ZIF-8/SrSe to consume GSH through DTNB fading was specifically observed in the ZIF-8/SrSe + GSH + DTNB group, as depicted in Figure 3B, C. Notably, we also assessed the glutathione oxidase activity of ZIF-8/Sr and ZIF-8/Se separately, and found no significant enzymatic activity. Significant glutathione oxidase-like activity was demonstrated solely by ZIF-8/SrSe, as evidenced in Figure 3D. We assessed the interaction of ZIF-8/SrSe at a concentration of 100 μg/mL with a gradient of GSH concentrations (1 to 6 mM) and a constant concentration of DTNB (100 μM). Figure 3E highlights a direct correlation between the absorbance at 412 nm and both time and GSH concentration, suggesting efficient GSH depletion by ZIF-8/SrSe. Meanwhile, the Km (Michaelis constant) and Vmax (Figure S4A) of the reaction catalyzed by ZIF-8/SrSe were determined using Lineweaver-Burk plots (Figure S4B), and the Km and Vmax with GSH as substrate were 0.301 mmol L^-1^ and 2.097 × 10 ^-5^ M s^-1^, respectively. In addition, the formation of hydrogen peroxide (H_2_O_2_) was monitored using an Amplex Red assay, the results of which are presented in Figure 3F. A consistent increase in fluorescence intensity at 520 nm with escalating concentrations of ZIF-8/SrSe (Figure 3G) was observed, substantiating the oxidase-mimicking activity exhibited by ZIF-8/SrSe. Drawing upon the four-electron (4e^-^) reduction theory previously documented in the literature 37, 38, we postulated that ZIF-8/SrSe might enable the reduction of oxygen to hydrogen peroxide (H_2_O_2_), in turn facilitating the generation of hydroxyl radicals (•OH). These radicals could then drive the catalytic oxidation of GSH to GSSG. The generation of •OH radicals in the presence of H_2_O_2_ and ZIF-8/SrSe was further substantiated by electron spin resonance (ESR) analysis, as illustrated in Figure 3H. Notably, the generation of •OH radicals was significantly enhanced in acidic conditions compared with weaker acidic or neutral environments, underscoring the potential applicability of ZIF-8/SrSe in therapies targeting the acidic microenvironment of tumors. Moreover, ultrahigh-performance liquid chromatography (UPLC) was employed to precisely quantify GSH and GSSG. The study findings indicated that GSH was oxidized to GSSG following the administration of ZIF-8/SrSe, as depicted in Figure 3I.

Moreover, during the metabolic processes of the body, superoxide radicals (O_2_·^-^) are continuously generated, especially in tumor cells with abnormal metabolism. Superoxide dismutase (SOD) is a critical enzyme that catalyzes the dismutation of O_2_·^-^ to H_2_O_2_. Therefore, SOD mimics can act as H_2_O_2_ producers in the tumor microenvironment (TME) 39. To evaluate the SOD-like activity of ZIF-8/SrSe nanozymes, we initially employed a nitroblue tetrazolium (NBT) chromogenic assay. In this assay, the interaction of L-methionine and riboflavin under UV light generated O_2_·^-^, which subsequently reduce NBT to formazan, evidenced by a distinct peak at 560 nm. We noted that as the concentration of ZIF-8/SrSe nanozymes increased, there was a decrease in absorbance intensity and a rise in the SOD inhibition rate, underscoring the nanozymes' potent SOD-mimicking activity (Figure 3J, k). Following this, the study explored ZIF-8/SrSe's potential to catalyze the oxidation of GSH in the presence of H_2_O_2_, using DTNB as a colorimetric indicator. It was observed that the increase in the level of ZIF-8/SrSe was directly proportional to the decrease in the absorbance at 412 nm, indicating reaction progress (as indicated in Figure 3L). Importantly, this catalytic reaction was successfully carried out under an oxygen-free N_2_ and Ar_2_ atmosphere, confirming the glutathione peroxidase activity of ZIF-8/SrSe and its effective consumption of GSH (Figure 3M). The quantitative evaluation of enzymatic kinetics confirmed that ZIF-8/SrSe conforms to the established Michaelis-Menten kinetics within the DTNB colorimetric assay, as shown in Supplementary Figure S5. In summary, the collective findings suggest that ZIF-8/SrSe manifests enzymatic activity analogous to that of GSH and harbors the potential to promote ferroptosis in tumor cells via the depletion of GSH.

### DFT Studies on the Glutathione Oxidase-like Activities of ZIF-8/SrSe

**Figure 4:**
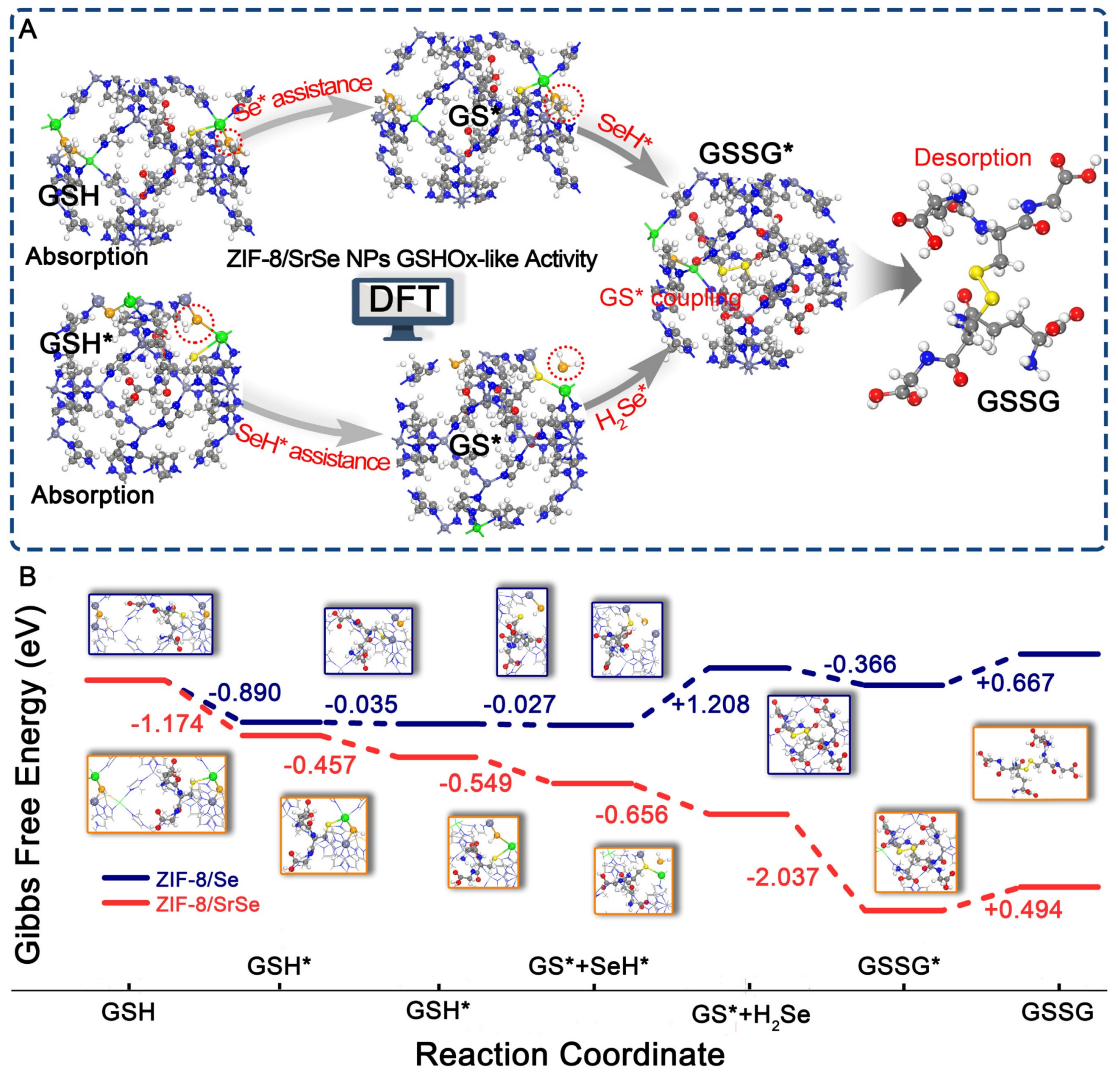
(A) Proposed GSHOx-like activity catalytic mechanism and B) free energy diagrams of ZIF-8/SrSe. Atom representation: Sr (green), Se (orange), N (blue), C (gray), O (red), H (white) and S (yellow).

To elucidate the potential catalytic mechanism of the ZIF-8/SrSe nanozyme's in GSHOx-like activity, we conducted Density Functional Theory (DFT) calculations to investigate its atomic-level catalytic behavior (Figure S6). As illustrated in Figure 4A, GSH initially approaches the Sr-Se-Zn site. The adsorbed GSH* interacts with Sr-Se-Zn, leading to the formation of GS* and Sr-SeH-Zn. The resulting Sr-SeH-Zn site can further react with the adsorbed GSH*. Simultaneously, the adsorbed GSH* and Sr-SeH-Zn interact, producing GS* and H_2_Se*. Finally, the two generated GS* intermediates undergo a coupling reaction to yield GSSG. To further investigate the GSHOx-like activity mechanism of ZIF-8/SrSe, we compared it with ZIF-8/Se without Sr doping (Figure 4B). During GSH adsorption, ZIF-8/SrSe exhibits a two-step distinct exothermic process with a total exothermicity of -1.631 eV. In contrast, the exothermicity of this process is significantly smaller for ZIF-8/Se, measuring -0.925 eV. Subsequently, the dehydrogenation of GSH* to GS* remains a significantly exothermic process on ZIF-8/SrSe, with energy changes of -0.549 eV and -0.626 eV, respectively. Conversely, the dehydrogenation of the first GSH* molecule on ZIF-8/Se is nearly non-exothermic (-0.027 eV), and the dehydrogenation of the second GSH* molecule exhibits a significantly endothermic process (+1.208 eV). Furthermore, the coupling reaction of GS* is exothermic on both ZIF-8/SrSe and ZIF-8/Se, with energy changes of -2.037 eV and -0.366 eV, respectively. Notably, the coupling reaction is more favorable on ZIF-8/SrSe. Overall, during the GSHOx-like activity process, the total Gibbs free energy of ZIF-8/SrSe is significantly reduced by -4.380 eV (Figure 4B), while that of ZIF-8/Se is +0.557 eV. The DFT calculations clearly demonstrate that ZIF-8/SrSe exhibits higher-performance GSHOx-like enzymatic activity compared to ZIF-8/Se, indicating that the Sr doping imparts enhanced enzymatic activity to ZIF-8/Se, consistent with our other experimental findings (Figure 3D).

### Response degradation and cellular internalization

**Figure 5:**
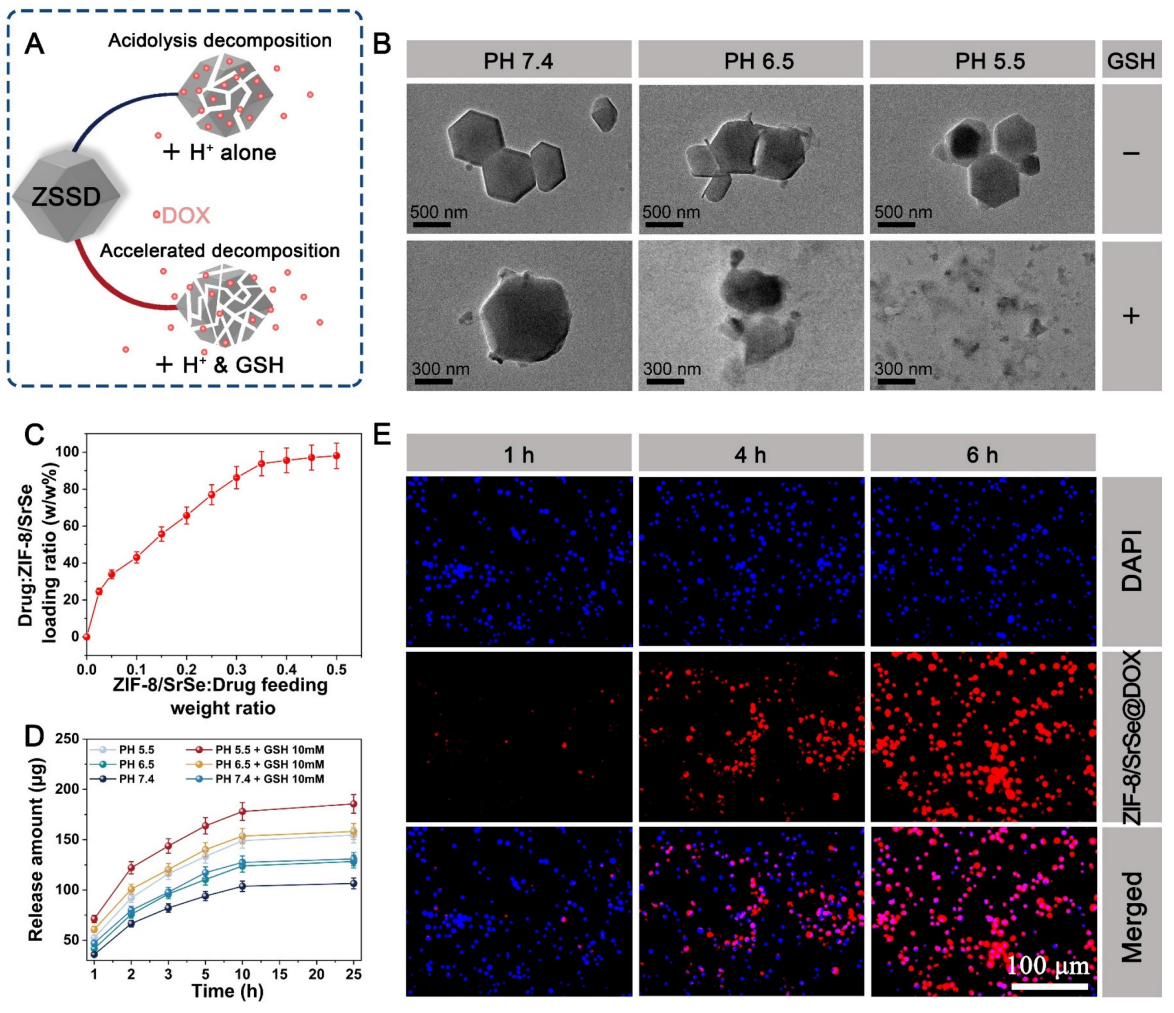
(A) Schematic representation of GSH/acid-responsive degradation of ZIF-8/SrSe@DOX. (B) TEM images of ZIF-8/SrSe@DOX dispersed in pH 5.5, 6.5, and 7.4 buffers with or without GSH (10 mM) for 24 hours. (C) DOX-loading weight ratios in ZIF-8/SrSe@DOX at different feeding ratios of ZIF-8/SrSe:DOX. (D) Release profile of DOX from ZIF-8/SrSe@DOX under different stimulation conditions (n=3). (E) Fluorescence microscopy images of H22 cells incubated with ZIF-8/SrSe@DOX at specific time points (1, 4, 6 hours). Blue and red represent DAPI and DOX fluorescence, respectively. Scale bars: 100 μm.

ZIF-8 nanoparticles have been widely utilized as drug carriers for loading various molecules, enzymes, DNA, and proteins, enabling pH-responsive controlled release 40. In Figure 5A, we observed that the formed ZIF-8/SrSe@DOX exhibited responsiveness to both GSH and acidic conditions, leading to their degradation and subsequent release of DOX. To investigate the decomposition behavior of ZIF-8/SrSe@DOX, we incubated them with PBS at different pH values (7.4, 6.5, and 5.5) in the presence or absence of GSH, and captured their morphological changes using TEM imaging (Figure 5B). Under pH 7.4 buffer conditions, the morphology of ZIF-8/SrSe remained largely intact, with slight degradation observed at the edges of the nanomaterials in the presence of GSH. However, a more noticeable degree of degradation was observed under pH 6.5 and pH 5.5 conditions, indicating the acid-responsive degradation behavior of the nanoparticles. Notably, in the presence of GSH, the degradation of ZIF-8/SrSe nanozyme was further enhanced, leading to fragmentation of their shape. These findings suggest that ZIF-8/SrSe nanozyme can selectively respond to the acidic tumor microenvironment characterized by overexpression of GSH, facilitating their targeted degradation.

The loading and releasing behavior of DOX in the ZIF-8/SrSe@DOX system was also investigated. As the feeding weight ratio of ZIF-8/SrSe to DOX increased, the loading efficiency of DOX gradually improved, reaching a maximum level of 94% (Figure 5C). Subsequently, we investigated the release profile of DOX from ZIF-8/SrSe@DOX in solutions with pH values of 7.4, 6.5, and 5.5, representing the microenvironment of normal tissues, tumor tissues, and lysosomes in tumor cells, respectively. The percentage of DOX released was determined using its standard curve (Figure S7). Interestingly, we observed an enhanced release of DOX in the presence of GSH and acidic conditions. These findings suggested a stimulation-responsive release pattern for DOX, which correlated with the degradation behavior of the ZIF-8/SrSe nanozyme. The cellular internalization of ZIF-8/SrSe@DOX was examined using fluorescence microscopy. H22 cells were treated with ZIF-8/SrSe@DOX, and the intracellular fluorescence intensity from DOX was tracked. Figure 5E demonstrates that the fluorescence from DOX within the cells increased incrementally, reaching its zenith at six hours, indicative of the nanozyme's successful cellular uptake and the effective transport of DOX into the nuclei of the H22 cells, which is essential for chemotherapy. For potential clinical application, the stability of the synthesized materials in biological environments is paramount. As shown in Figure S8, the ZIF-8/SrSe nanozyme displayed exceptional stability when suspended in water, phosphate-buffered saline (PBS), and cellular culture medium over a period of 72 hours, confirming its robust biostability.

### Antitumor effect and mechanism analysis of ZIF-8/SrSe@DOX *in vitro*

**Figure 6:**
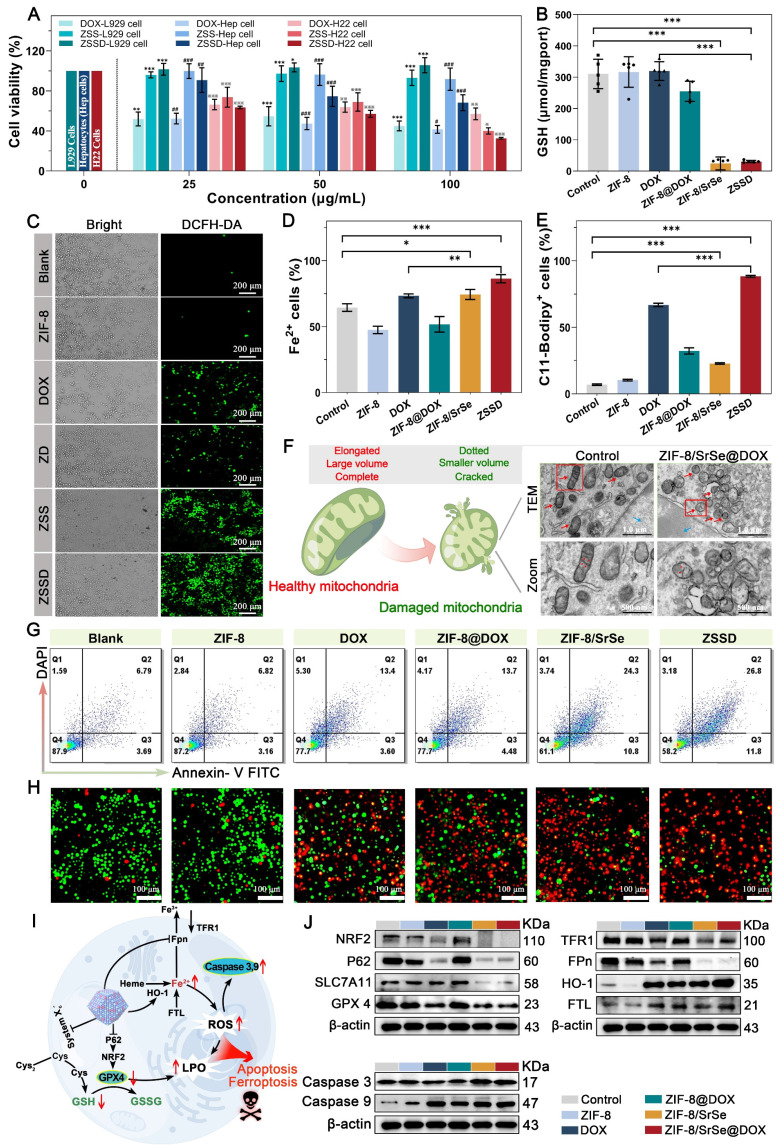
** Antitumor and mechanism analysis of ZIF-8/SrSe@DOX in vitro.** (A) Effect of DOX, ZIF-8/SrSe and ZIF-8/SrSe@DOX on L929, primary hepatocytes (Hep cells), and H22 cell viability (n=5). (B) The content of GSH was measured in ZIF-8, DOX, ZIF-8@DOX, ZIF-8/SrSe and ZIF-8/SrSe@DOX-treated H22 cells (n=5). (C) Fluorescence images of DCFH-DA-stained H22 cells after exposure to ZIF-8, DOX, ZIF-8@DOX, ZIF-8/SrSe and ZIF-8/SrSe (25 μg·mL^-1^) for 12 h. (D) Flow cytometry (FACS) for cellular Fe^2+^ in ZIF-8, DOX, ZIF-8@DOX, ZIF-8/SrSe and ZIF-8/SrSe@DOX (25 μg·mL^-1^ for 12 h)-treated H22 cells stained with Far-Red labile Fe^2+^ fluorescence probe (n=3). (E) FACS for lipid ROS in ZIF-8, DOX, ZIF-8@DOX, ZIF-8/SrSe and ZIF-8/SrSe@DOX (25 μg·mL^-1^ for 12 h)-treated H22 cells stained with CD11-BODIPY lipid probe (n=3). (F) TEM image of H22 cells upon ZIF-8/SrSe@DOX NPs (100 μg·mL^-1^) treatment for 12 h. The blue and red arrows indicate the positions of the nucleus and mitochondria, respectively. (G) FACS analysis results of H22 cell apoptosis after treatment in different experimental groups (n=3) and H) fluorescence images of Calcein-AM (live/green) and PI (dead/red) co-stained cells. (I) Western blot for ferroptosis/apoptosis-related pathways in materials-treated H22 cells. (J) Quantification of related proteins expression level in materials-treated H22 cells. (*P < 0.05, **P < 0.01, ***P < 0.001).

Motivated by the potent anti-cancer properties of DOX and the GSH elimination capability of ZIF-8/SrSe nanozyme, we investigated the ability of ZIF-8/SrSe@DOX to induce apoptosis and ferroptosis *in vitro*. Initially, we evaluated the cytotoxicity of DOX, ZIF-8/SrSe, and ZIF-8/SrSe@DOX against L929 cells, primary hepatocytes (Hep cells), and H22 cells using a CCK-8 assay (Figure 6A). After a 12-hour incubation period, the cell viability of L929, Hep, and H22 cells in the DOX groups all decreased to approximately 50%, indicating the consistent cytotoxic effect of DOX across these cell types. When ZIF-8/SrSe or ZIF-8/SrSe@DOX were evaluated, both exhibited good biocompatibility with normal cells, showing only slight cytotoxicity towards L929 and Hep cells. However, for H22 cells, the cell viability after incubation with DOX and ZIF-8/SrSe (100 µg mL^-1^) was 57.07 % and 40.07 %, respectively. Remarkably, the combined treatment of DOX and ZIF-8/SrSe resulted in a further reduction in cancer cell viability to 32.66 %, benefiting from the synergistic effect of DOX chemotherapy and the catalytic degradation therapy (CDT) provided by ZIF-8/SrSe nanozyme. These results demonstrate that introducing ZIF-8/SrSe@DOX significantly suppressed the viability of H22 cells, indicating the specific therapeutic effect of the materials against tumor cells. This selectivity is likely attributed to the distinct intracellular microenvironments between normal and tumor cells. Moreover, this promising selectivity can enhance the biosafety profile of ZIF-8/SrSe@DOX nanoparticles.

Based on their good cytotoxic effect, further exploration of the internal therapeutic mechanism of ZIF-8/SrSe@DOX was launched. Firstly, to determine the type of ZIF-8/SrSe@DOX induced cell death, we treated H22 cells by using autophagy (3-Methyladenine, 3-MA) and necroptosis (Nec-1) inhibitors under ZIF-8/SrSe@DOX administration conditions. Interestingly, the presence of 3-MA or Nec-1 did not counteract the decrease in cell viability observed in H22 cells treated with ZIF-8/SrSe@DOX (as shown in Figure S9A), suggesting that ZIF-8/SrSe@DOX does not induce autophagy or necrosis. Considering that GSH depletion can initiate cell ferroptosis 41, we then exposed H22 cells to both a ferroptosis activator (RSL3) and an inhibitor (Ferrostatin-1, Fer-1). We observed that the addition of RSL3 further reduced the viability of ZIF-8/SrSe@DOX-treated H22 cells, suggesting a role for ferroptosis in the mechanism of cytotoxicity. The incorporation of Fer-1 substantially mitigated the cell death in H22 cells triggered by ZIF-8/SrSe@DOX, as evidenced in Figure S9B. This result implies that ZIF-8/SrSe@DOX is capable of inducing ferroptosis in H22 tumor cells. Furthermore, we evaluated the depletion of GSH triggered by ZIF-8/SrSe@DOX in H22 cells. As depicted in Figure 6B, there was a significant diminution in GSH levels following the treatment with ZIF-8/SrSe nanozyme and ZIF-8/SrSe@DOX. This decrease is likely possibly attributable to the oxidative conversion of GSH to GSSG, mediated by the GSH oxidase-mimetic activity of the ZIF-8/SrSe nanozyme. The absence of significant glutathione depletion in ZIF-8/Sr and ZIF-8/Se alone further suggests that the doping of Sr results in superior enzymatic activity (Figure S10). As has been reported, depletion of GSH may cause the accumulation of lipid peroxides (LPO), which is an indicator of ferroptosis 24*.* As predicted, ZIF-8/SrSe@DOX addition induced large amounts of LPO production, as evidenced by the rise in malondialdehyde (MDA) concentration in H22 cells (Figure S11). Considering that GSH depletion often precipitates oxidative stress, we monitored the generation of intracellular ROS utilizing 2',7'-dichlorodihydrofluorescein diacetate (DCFH-DA) staining. Concordant with our expectations, treatment with ZIF-8/SrSe@DOX elicited robust green fluorescence in H22 cells (Figure 6C), signifying a marked elevation in the total intracellular ROS levels (Figure S12). This increase is likely attributable to the generation of •OH through a Fenton-like reaction facilitated by the ZIF-8/SrSe@DOX complex 24. Fe^2+^ levels in H22 cells were assessed using the Far-Red Fe^2+^ fluorescent probe.

Upon exposure to ZIF-8/SrSe@DOX, a conspicuous increase in Fe^2+^ content was observed (Figure 6D). These findings imply that ZIF-8/SrSe@DOX treatment may lead to an accumulation of free iron within H22 cells, potentially triggering the generation of ROS through a Fenton-like reaction 42. Supporting this, a similar upsurge in lipid ROS levels, indicated by the ferroptosis marker C11-BODIPY, was noted in the material-treated H22 cells (Figure 6E). Furthermore, the critical role of mitochondrial morphology as a determinant for ferroptosis was considered, and the occurrence of ferroptosis was substantiated through an analysis of mitochondrial ultrastructure. Figure 6F and Figure S13 demonstrate that exposure to ZIF-8/SrSe@DOX induced a pronounced morphological shift in mitochondrial structure from an elongated to a dotted form. This observation indicated mitochondrial membrane condensation and a reduction in mitochondrial volume.

TEM imaging of H22 cells treated with ZIF-8/SrSe@DOX revealed pronounced chromatin condensation, a characteristic feature of apoptosis, shown in Figure 6F and Figure S13. The use of the ferroptosis inhibitor, Fer-1, was unable to fully restore viability to H22 cells treated with ZIF-8/SrSe@DOX, as shown in Figure S9B, suggesting that the ZIF-8/SrSe@DOX may also induce cell death via pathways other than ferroptosis. Furthermore, treatment with ZVAD-FMK, a known apoptosis inhibitor, led to partial recovery of cell viability in ZIF-8/SrSe@DOX-treated H22 cells (Figure S14). Indicating that the ZIF-8/SrSe@DOX nanozyme has the potential to promote apoptosis in H22 cells. Clinically, DOX is recognized for its role in inducing apoptosis through inhibiting DNA synthesis 43. Thereupon, the apoptosis of different formulations was further investigated by the Annexin V-FITC/DAPI apoptosis assay. After incubation with ZIF-8, DOX, ZIF-8@DOX, ZIF-8/SrSe and ZIF-8/SrSe@DOX, the apoptosis percentage of H22 cells in the DOX, ZIF-8@DOX and ZIF-8/SrSe group was 16.7 %, 18.18% and 35.1%, respectively (Figure 6G, S15), demonstrating that DOX and ZIF-8/SrSe both can induce apoptosis.

Moreover, we found that the ZIF-8/SrSe@DOX group showed a relatively higher apoptosis rate 38.6%, which was higher than other groups, indicating that the combination of chemotherapy and CDT was more effective than each individual treatment alone. This conclusion was further substantiated by the calcein-AM and propidium iodide (PI) co-staining assays—where calcein-AM marks live cells with green fluorescence and PI indicates dead cells with red fluorescence—as shown in Figure 6H. The synergistic treatment of chemotherapy/CDT (ZIF-8/SrSe@DOX group) led to the highest number of dead cells in H22 cultures when compared with the control group, the group treated with chemotherapy alone (DOX group), or the group treated with CDT alone (ZIF-8/SrSe group).

To further elucidate the mechanisms through which ZIF-8/SrSe@DOX induces ferroptosis and apoptosis in tumor cells, we analyzed ferroptosis- and apoptosis-related pathways in pretreated H22 cells. The role of iron accumulation and ensuing lipid peroxidation is pivotal in precipitating ferroptosis 44; therefore, molecules and signaling pathways involved in iron homeostasis and lipid peroxidation are crucial regulators of this process 45. Firstly, iron uptake, translocation and storage all exert some regulatory effect on ferroptosis. As shown in (Figure 6I, J and S16), after DOX and ZIF-8/SrSe nanozyme treatment, the cellular uptake of the iron membrane protein transferrin receptor 1 (TFR1) decreased, while the iron storage protein (FTL) increased. This indicated that the cells contained abundant iron, including Fe^2+^_,_ thereby increasing their risk of ferroptosis in the cells. In addition, the ZIF-8/SrSe-treatment inhibited the expression of iron ion exporter (Fpn), and exposure to the ZIF-8/SrSe nanozyme increased Fe^2+^ content as described above. Given that Fpn is the exclusive iron efflux channel in cells, the inhibition of Fpn leads to increased intracellular the Fe^2+^ accumulation, which in turn catalyzes the production of ROS, ultimately exacerbating the ferroptosis process 46. Exposure to DOX and ZIF-8/SrSe nanozyme significantly upregulated HO-1 protein expression in H22 cells. Ordinarily, increased HO-1 expression assists cells in mitigating redox stress 47, However, chronic upregulation of HO-1 leads to the induction of ferroptosis 48. The enzymatic action of HO-1 converts heme into carbon monoxide, biliverdin, and Fe^2+^. The overabundance of Fe^2+^ within the cells that serves as a direct provocateur of ferroptosis 49. As ROS levels continue to rise, cancer cells need to prevent further increases to maintain ROS at a certain level, and therefore elevate intracellular antioxidant systems such as GSH to achieve an intracellular balance. We found that the GSH content in the ZIF-8/SrSe nanozyme and ZIF-8/SrSe@DOX-treated H22 cells was significantly decreased (Figure 6B). GSH acts as a vital cofactor for GPX4. The depletion of GSH potentially leads to the inactivation of GPX4 in tumor cells, an event associated with the accumulation of lipid peroxides (LPOs), an outcome that facilitates ferroptosis. Consequently, we analyzed the intracellular expression levels of GPX4 after treatment with different formulations using Western blotting to elucidate this mechanism. As shown in (Figure 6I, J and S16), there was an obvious decline of GPX4 expression in nano-formulations that contained DOX and ZIF-8/SrSe, indicating that ZIF-8/SrSe@DOX can effectively deplete GSH to inactivate GPX4. SLC7A11 is a transmembrane protein that regulates the transport of cysteine (a precursor amino acid for GSH biosynthesis) and has a key role in maintaining the activity of the GPX4/GSH antioxidant system 50. As expected, the ZIF-8/SrSe nanozyme reduced SLC7A11 protein levels, thereby inhibiting cysteine uptake and reducing GSH synthesis, and GSH depletion and inhibition of synthesis led to toxic peroxide accumulation, protein and cell membrane damage, and subsequent cellular ferroptosis 51. The SLC7A11-GSH-GPX4 axis is considered the major cellular system that promotes ferroptosis. Moreover, the use of DOX and ZIF-8/SrSe nanozyme also decreased the expression of NRF2 and its regulating protein P62 expression (Figure 6I, J and S16) As we know that NRF2 is a master regulator of the antioxidant response, and its inhibition increases cellular uptake of iron and promotes ROS production, therefore, NRF2 can promote the occurrence of ferroptosis. Additionally, the reduction of the proto-oncoprotein p62 promoted NRF2 degradation and subsequently the transcriptional activity of NRF2 was further inhibited to promote ferroptosis 52. Collectively, these results demonstrated that the ZIF-8/SrSe nanozyme induce ferroptosis via a GSH depletion dependent ROS production mechanism, while DOX gives a synergistic effect during ZIF-8/SrSe@DOX-induced ferroptosis. Concurrently, immunoblot analysis data provided compelling evidence of the upregulation of cleaved Caspase 3 and Caspase 9 expression in the ZIF-8/SrSe@DOX group compared to the blank PBS group (Figure 6I, J and S16), validating the ability of the developed ZIF-8/SrSe@DOX nanoparticles to induce apoptosis. These findings unequivocally demonstrated the involvement of both ferroptosis and apoptosis in the mechanism of tumor cell death induced by ZIF-8/SrSe@DOX.

### Antitumor assay and biosafety *in vivo*

**Figure 7:**
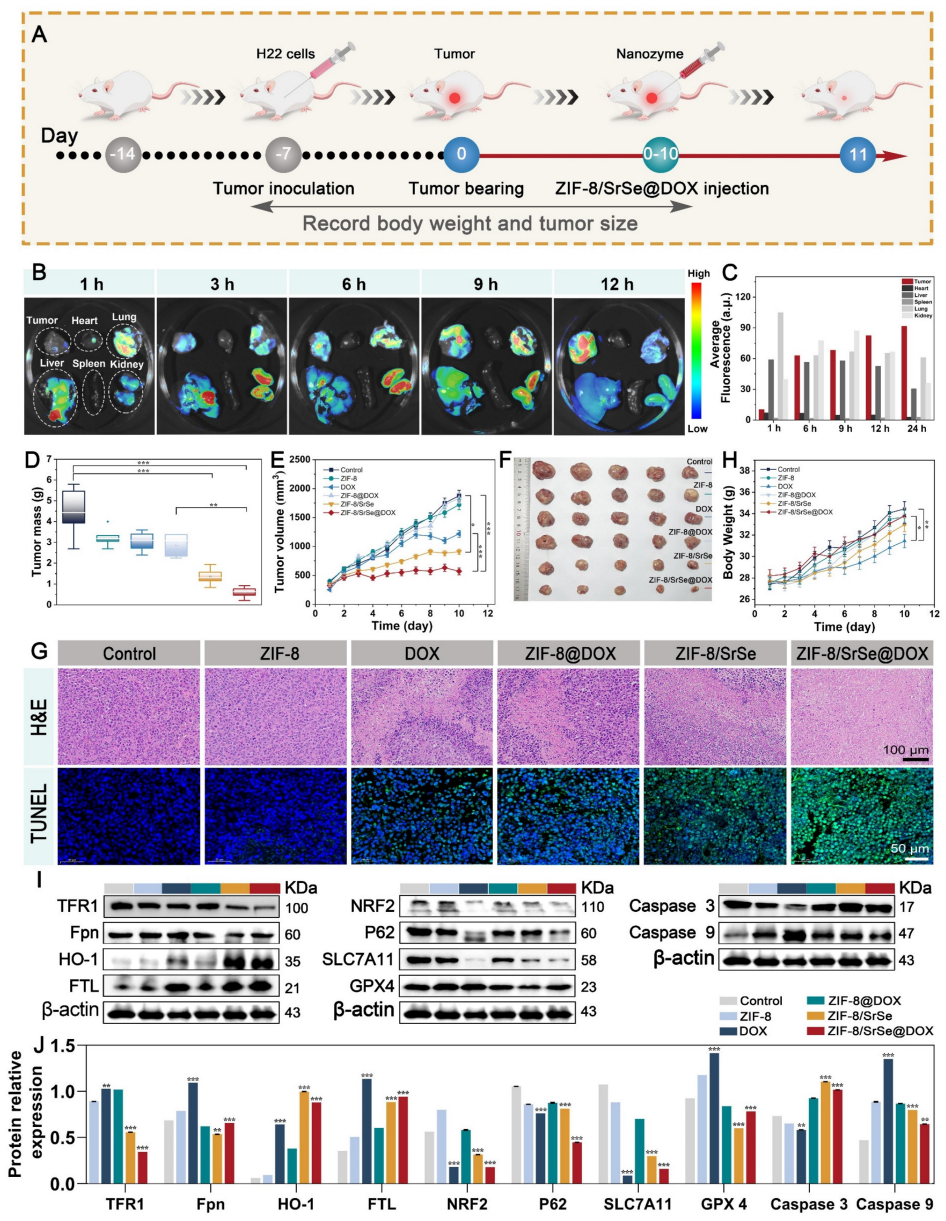
(A) Schematic illustration of the typical therapeutic procedures. (B) Ex. vivo fluorescence images of major organs (Heart, Lung, Liver, Spleen, Kidney) and tumor dissected from mice at 1, 3, 6, 9, 12h after injected with ZIF-8/SrSe@DOX NPs. (C) Semi-quantitative fluorescence intensities of various organs and tumor which determined at different time points. (D) Tumor mass and E) tumor volume curves after the mice recelved different treatments. (F) Pictures of tumors dissected on the 11th day after different treatments. (G) Representative histological images of H&E and TUNEL immunofluorescence staining of mouse tumor tissue sections after different treatments. (Scale bar: 100 μm, 50 μm). (H) Body weight curves of the H22 tumor-bearing mice after different treatments. (I) Western blot for ferroptosis/ apoptosis-related pathways in tumor tissue. (J) Quantification of related proteins expression level in materials-treated cancer tissue (*P < 0.05, **P < 0.01, ***P < 0.001).

After the synergistic therapeutic effect was observed in vitro, the in vivo antitumor effect of ZIF-8/SrSe@DOX was investigated in H22 tumor-bearing mice. Once the tumor sizes reached approximately 65 mm^3^, the therapeutic intervention was initiated, and the experimental procedure is visually depicted in Figure 7A. The mice were divided into six groups and given different treatments: saline, ZIF-8, DOX, ZIF-8@DOX, ZIF-8/SrSe, and ZIF-8/SrSe@DOX, respectively. Initially, the accumulation of ZIF-8/SrSe@DOX at the tumor site in mice was investigated through fluorescence imaging after intravenous injection. The mice were euthanized at various time intervals, and the tumor as well as major organs were collected and imaged. The fluorescence signal in the tumor region was observed to gradually intensified following injection (Figure 7B), affirming the effective localization of ZIF-8/SrSe@DOX at the tumor site. Quantitative analysis of the fluorescence images obtained from the harvested tissues revealed, revealing a significantly higher fluorescence signal in the tumor than in the major organs, thus further confirming the nanazyme remarkable accumulation ability (Figure 7C). The changes in tumor weight and volume of the mice throughout the 10-day treatment period are presented in (Figure 7D, E), respectively. Compared with the control group, all other groups showed different degrees of anti-tumor effects. Notably, ZIF-8/SrSe could effectively inhibit the growth trend of tumors, but this inhibition was more pronounced in the ZIF-8/SrSe@DOX group. This supports the notion that chemodynamic-chemotherapeutic combination therapy is superior to monotherapy. This observation is further supported by the digital photographs of the tumors taken at 10 days post-treatment, as shown in Figure 7F. The ZIF-8/SrSe@DOX group exhibited a pronounced preferential tumor suppression effect. The therapeutic efficacy of the nanomaterials was verified at the cellular level using hematoxylin and eosin (H&E) as well as TdT-mediated dUTP nick end labeling (TUNEL) staining. As shown in Figure 7G and S17, the ZIF-8/SrSe@DOX group had tumor sections with the greatest damage, whereas apoptosis was almost negligible in the control group. These results highlight the superiority of the synergistic treatment approach. Additionally, the mice's body weight was monitored to estimate the formulation's biosafety. Figure 7G illustrates that mice treated with saline, ZIF-8@DOX, ZIF-8/SrSe, and ZIF-8/SrSe@DOX did not exhibit significant changes in body weight over the 10-day treatment period, indicating the absence of acute toxicity associated with our synergistic treatment strategy.

In contrast, administration of free DOX resulted in a slow weight gain in mice, indicating systemic toxicity. Furthermore, H&E staining images of major tissues and organs (Figure S18) as well as various biochemical indices (Figure S19, S20 and Table S2) from the ZIF-8/SrSe and ZIF-8/SrSe@DOX groups revealed no significant pathological abnormalities after 10 days of treatment. However, treatment with DOX alone caused mild heart and hepatic damage, including a sharp reduction in liver tissue weight (Figure S21), further confirming the favorable *in vivo* biocompatibility of ZIF-8/SrSe@DOX. Compared to free DOX, ZIF-8/SrSe@DOX had a lower impact on different organs, including the heart, liver, spleen, lung, and kidney. The expression of ferritin-related genes, such as GPX4, in tumor tissues was assessed through western blotting (Figure 7I, J). It was observed that ZIF-8/SrSe@DOX significantly affected iron accumulation and reduced lipid peroxidation, consistent with the results obtained from the *in vitro* experiments. The levels of regulated GPX4 and SLC7A11 were notably diminished (Figure 7I, J). These findings further support the ability of ZIF-8/SrSe@DOX possess the ability to induce ferroptosis via a mechanism involving GSH depletion-dependent ROS production, thereby demonstrating its potent anticancer properties.

To ensure the practical applicability of nanomaterials, it is crucial to thoroughly investigate their biological toxicity. Thus, we conducted an extensive systematic study on the safety of ZIF-8/SrSe@DOX in healthy mice. Hemolysis, a critical factor for *in vivo* applications, was assessed to confirm the biocompatibility of the synthesized materials. The hemolysis test revealed no evident hemolysis in mouse erythrocytes treated with ZIF-8/SrSe@DOX (Figure S222). Furthermore, to evaluate the potential toxicological effects of ZIF-8/SrSe@DOX during treatment, healthy mice were intravenously injected with these nanoparticles and sacrificed at 7 and 28 days. Biological samples were collected for analysis. The body and major organ weights of mice injected with ZIF-8/SrSe@DOX at 7 and 28 days did not exhibit significant abnormalities compared to the control group (Figure S23). Biochemical and hematological parameters were within the normal range and similar to those of the control group (Figure S24). These findings indicate that ZIF-8/SrSe@DOX did not induce notable hepatotoxicity or renal dysfunction. Additionally, histological analysis of H&E-stained sections from all organs showed similar morphological features between the treatment groups (Figure S25), indicating the absence of tissue or cellular damage.

## Conclusion

In conclusion, our study reveals an integrative strategy for the potent ablation of tumors via concurrent chemotherapy and CDT. This approach employs an innovative DOX-loaded ZIF-8/SrSe nanozyme that pairs chemotherapeutics with a Fenton reactant. The ZIF-8/SrSe@DOX achieves controlled biodegradation within the TME in the presence of GSH and acidic conditions, resulting in the targeted release of DOX and consequent induction of DNA damage, apoptosis, and partial ferroptosis in tumor cells. The nanozyme's composition, doped with Sr and Se, imparts robust GSH oxidase-like activity, thereby concurrently inactivating the GPX4 and SLC7A11 proteins. This study conducted a thorough investigation into its regulatory effect on iron metabolism, including iron uptake and transfer via TFR1 and FPN, manipulation of storage through FTH/L, and induction of HO-1. This disruption of the SLC7A11-GSH-GPX4 pathway and iron metabolism substantially encourages ferroptosis within tumor cells, evidenced both *in vitro* and *in vivo*. Additionally, the ZIF-8/SrSe@DOX complex markedly downregulates the NRF2 pathway and its associated protein P62, further promoting tumor cell ferroptosis. Together, these findings robustly support the potential of the ZIF-8/SrSe@DOX nanozyme as a promising therapeutic agent against cancer.

This study supports an important therapeutic strategy and theoretical basis for nano-enzyme-based cancer treatments. Although the research carried out so far is still a long way from actual clinical application, the in-depth and comprehensive basic research is a prerequisite for clinical application. We hope that our research will encourage the eventual clinical translation of nano-enzymes such as ZIF-8/SrSe@DOX.

## Supplementary Material

Supplementary materials and methods, figures and tables.
