# Supplementary material for: Multifunctional Sr/Se co-doped ZIF-8 nanozyme for chemo/chemodynamic synergistic tumor therapy via apoptosis and ferroptosis
Source: Theranostics. 2024 Feb 24;14(5):1939–55. doi: 10.7150/thno.92663 (PMC10945335; doi:10.7150/thno.92663)
Supplement: Supplementary file 1 — Supplementary materials and methods, figures and tables. [file thnov14p1939s1.pdf]

Supporting Information

Multifunctional Sr/Se co-doped ZIF-8 nanozyme for Chemo/Chemodynamic synergistic tumor therapy via Apoptosis and Ferroptosis

Aimin Wu[1,§], Ming Han[2,§], Zihan Ni[6], Haoran Li[3], Yinyin Chen[3], Zhouping Yang[1], Yumei Feng[4], Zufeng He[5], Hua Zhen[2], Xianxiang Wang[2*]

[1]*Institute of Animal Nutrition, Sichuan Agricultural University, Chengdu 611130, China*

[2]*College of Science, Sichuan Agricultural University, Chengdu 611130, Sichuan, China*

[3]*College of Agronomy, Sichuan Agricultural University, Chengdu 611130, Sichuan, China*

[4]*State Key Laboratory of Crop Gene Exploration and Utilization in Southwest China, Chengdu 611130, Sichuan, China*

[5]*Institute of New Rural Development, Sichuan Agricultural University, Chengdu 611130, Sichuan, China*

[6]*College Veterinary Medicine, Sichuan Agricultural University, Chengdu 611130, China*

[#] These authors contributed equally to this work.

*To whom correspondence should be addressed: Xianxiang Wang

E-mail address: xianxiangwang@hotmail.com.

Materials and Methods

Materials and reagents

Zinc nitrate hexahydrate Zn $(NO_3)_2·6H_2O$, Strontium acetate (Sr (O Ac)$_2$), Sodium selenite (Na_2SeO_3), Tris(hydroxymethyl), Methanol (Me OH), 2-Methylimidazole (2-MeIM), Hydrogen peroxide (H_2O_2, 30%), Ethylenediamine tetraacetic acid disodium salt (EDTA), Methionine (Met), Riboflavin, were supplied by KeLong (Chengdu, China, http://www.cdkelong.com/). Doxorubicin (DOX), Nitro blue tetrazolium (NBT), Glutathione (GSH), 5, 5'- Dithio bis-(2-nitrobenzoic acid) (DTNB), Amplex Red reagent was purchased from Macklin Biochemical Co. Ltd. (Shanghai, China, http://www.macklin.cn/). 10 x PBS (pH7.2-7.4, 0.1 mol L^{-1}) was bought from Beijing Soleibao Technology Co., Ltd. All chemicals used in the experiments were analytically pure and used without further purification was required. All experiments used deionized water (DW).

The BCA protein assay kit was obtained from TransGen (Beijing, China). Annexin V-FITC/PI apoptosis detection kit, Calcein-AM and propidium iodide (PI), 4',6-diamidino-2-phenylindole (DAPI), 2,7-dichlorofluorescein diacetate (DCFH-DA). Female mice aged 6 weeks were purchased from Chengdu Dashuo experimental animal Co., Ltd.

Characterization

The crystal structure, morphological structure, elemental composition, and other Properties were analyzed by X-ray diffraction (XRD, DX-2700, Dandong, China), Transmission Electron Microscope (TEM, JSM4800F, JEOL, Japan), Scanning Electron Microscope (SEM, JEOL-2100F, Japan), X-ray Photo electron Spectroscopy (XPS, ESCALAB-250Xi, China), Fourier Transform Infrared Spectrometer (FTIR, bruker vertex 70, China). Fluorescence spectrophotometer (Hitachi, F-4500, Tokyo, Japan). BET surface area and pore volume of prepared material were measured from nitrogen adsorption-desorption isotherms via a NovaWin 1000e instrument (Quantachrome, USA). Electron Spin Resonance (ESR) experiments were conducted on the Bruker A300 spectrometer. UV-*vis* absorption spectra were obtained by UV-*vis*

spectrophotometer (Aoyi instrument Co. Ltd., A390, shanghai, China).

Synthesis of ZIF-8/SrSe with encapsulated DOX

In a typical synthesis of ZIF-8, 5 g of 2-methylimidazole was dissolved in 50 mL of methanol, which was subsequently added into 50 mL of methanol containing 2.35 g of Zn $(NO_3)_2 \cdot 6H_2O$ with stirring for 24 h at room temperature, and the mixture became milky. The final products were collected by centrifugation (10000 rpm, 10min), and washed with methanol for several times and dried at 70 °C in vacuum oven overnight. 0.2 g of as-prepared ZIF-8 and 0.12 g (1 mmol) of Tris(hydroxymethyl) aminomethane were dispersed in 100 mL of a mixture (ethanol/ DI water, 1/1) by sonication and stirring to form solution A. 0.3 g (1.5 mmol) of Strontium acetate, 0.129 g (1.5 mmol) of Sodium selenite, and 0.06 g NaBH4 (1.5 mmol) were dissolved in 100 mL of DI water to form solution B. Then solution B was added into solution A. Followed by vigorous magnetic stirring for 12 h at room temperature, the product was collected via centrifugation and washed with DI water and ethanol for several times, and finally dried at 70 °C in vacuum oven overnight. Finally, DOX was loaded into the Porosity of ZIF-8/SrSe as follows: different concentrations of DOX solution were mixed with 0.2 mg/mL ZIF-8/SrSe solution, respectively, and stirred for 12 h in the dark to obtain ZIF-8/SrSe@DOX.

DFT calculation

DFT calculation of the energy change for the replacement of Zn $(Im)_2$ with Sr $(Im)_2$:

For M3(BTC)2 cluster model, all DFT calculations were carried out using Becke three parameters hybrid exchange-correlation functional (B3LYP) density functional implemented in Gaussian 16. The Stuttgart-Dresden basis set-RECP (SDD) was employed for Zn and Sr atoms, and the 6-31g(d) basis set was employed for C, H, and N atoms for the optimization of geometries and the calculation of the potential energies [1-3]. M(2-methylimidazole)2, where M is Zn or Sr cation, was used as the calculation model for the interaction between the 2-methylimidazole anion and the M cation.

DFT computational study on the catalytic reaction mechanism of GSH in SrSe@ZIF-8 model:

All DFT calculations were carried out using the CP2K code [4]. All calculations employed a mixed Gaussian and planewave basis sets. Core electrons were represented with norm-conserving Goedecker-Teter-Hutter pseudopotentials [5], and the valence electron wavefunction was expanded in a double-zeta basis set with polarization functions [6] along with an auxiliary plane wave basis set with an energy cutoff of 360 eV. The generalized gradient approximation exchange-correlation functional of Perdew, Burke, and Enzerhof (PBE) [7] was used. Each configuration was optimized with the Broyden-Fletcher-Goldfarb-Shanno (BGFS) algorithm with SCF convergence criteria of 1.0×10^{-6} au. To compensate the long-range van der Waals dispersion interaction between the adsorbate and the MOF skeleton, the DFT-D3 scheme [8] with an empirical damped potential term was added into the energies obtained from exchange-correlation functional in all calculations.

GSH oxidase-like activity assay

GSH oxidase-like activity was assessed with different concentrations of GSH using DTNB as a substrate [9]. 250 μL of DTNB solution (PBS, 3.0 mg mL^{-1}) and 200 μL of GSH solution (H_2O, 10 mM) were mixed into 200 μL of ZIF-8/SrSe (5 mg/mL) aqueous solution. The absorbance of the supernatant was then measured. The yellow products of DTNB and GSH were recorded by spectrophotometer at 412 nm.

SOD-like Activity Assay

The SOD-mimicking activities of ZIF-8/SrSe nanozymes were evaluated by calculating the inhibition of the NBT photoreduction. Methionine (0.3 mL, 130 mM), riboflavin (0.3 mL, 200 μM), NBT (0.3 mL, 750 μM), and ZIF-8/SrSe (0.1 mL) with final concentrations of 0.05, 0.1, 0.2, 0.3,0.4, and 0.5 mg mL^{-1} was respectively added to PBS solution (0.2 M, pH 7.4) and fixed the final volume to 3 mL. The as-prepared reaction solutions were placed in a box wrapped in tin foil and irradiated by a UV lamp for 15 min, with the group not subjected to UV treatment as the control group. After irradiation, the measurement of absorbance at 560 nm was immediately carried out. The inhibition percentage was determined based on the following equation: inhibition (%) = $[(A_0-A)/A_0] \times 100\%$, where A is the absorbance of various samples and A_0 is the

absorbance of the control.

GPx-like Activity Assay

Glutathione (GSH) reacts with 5,5 ′-dithiondinitrobenzoic acid (DTNB) to form a compound with 412 nm characteristic absorption [10]. During the test, glutathione is oxidized to oxidized glutathione (GSSG) in the presence of H_2O_2. The glutathione peroxidase (GPx) activity of ZIF-8/SrSe was measured by DTNB direct method. The decrease of GSH concentration was directly proportional to the catalytic activity of ZIF-8/SrSe.

Amplex Red for the Generation of H_2O_2

To test the generation of H_2O_2, Amplex red was used as a probe. In brief, ZIF-8/SrSe (0, 0.5, 1.0, and 2.0 μg·μL^{-1}) with GSH (0.4 mM) were mixed with Amplex red (0.1 μg·μL^{-1}) in PBS buffer for 1 h. Fluorescence spectra of the mixture solution were recorded using a Fluorescence spectrophotometer (Hitachi, F-4500, Tokyo, Japan) at 480 nm (excitation) and 582 nm (emission).

Identification of Catalytic Products Based on UPLC

Catalytic products were determined with ultrahigh-performance liquid chromatography (UPLC)/high-resolution mass spectroscopy (ACQUITY UPLC H-Class). Chromatographic separation was carried out using a Waters ACQUITY BEH C18 column (2.1 × 100 mm i.d., 1.8 μm, Waters ACQUITY UPLC HSS T3, Milford, MA, USA) at 40 °C. A mobile phase consisting of 96% Phosphate buffer (A, 6.8g of sodium dihydrogen phosphate and 2.2g of sodium heptanesulfonate, add 1000ml of deionized water to dissolve and adjust the PH value to 3 with phosphoric acid) and 4% methanol (B) was delivered at a total flow rate of 0.3 mL·min^{-1}. The injection volume was 2 μL. Detection wavelength is 210nm.

Electron Spin Resonance (ESR) Assay

5,5-Dimethyl-1-pyrro-line-N-oxide (DMPO) was chosen as a trapping agent for •OH. In a typical test procedure, 20 μL of ZIF-8/SrSe (500 μg·mL^{-1}) was added into 1 mL of PBS buffer (pH = 5.5,6.5,7.4, respectively) containing 100 μM H_2O_2 and 200 μM DMPO for ESR (Elexsys E580-10/12, Bruker,Germany).

ZIF-8/SrSe Decomposition and DOX Responsive Releasing

ZIF-8/SrSe nanoparticles were de-incubated with different PH of PBS (5.5, 6.5 and 7.4) and in the presence and absence of GSH. At designated time, the solution was characterized by TEM and UV–vis spectrometer.

To investigate DOX releasing behavior, The formed ZIF-8/SrSe@DOX NSs was sealed in the dialysis bag and put in buffers with different pH values at 7.4, 6.5, or 5.5, with or without GSH (10 mM). The buffer solutions outside the dialysis bag were collected at different time points of 1, 2, 3, 5, 10 and 24 h. The corresponding absorbance of DOX was detected by UV-vis-NIR spectrophotometry.

Cellular Uptake/Release Experiment

For ZIF-8/SrSe@DOX uptake, H22 cells were plated in 12-Well PCR Plates at 1.0×10^5 per well. Then, cells were treated with 1 mL of RPMI-1640 medium (10% FBS) containing ZIF-8/SrSe@DOX (10 μg) for 1, 2, 4 and 6h. After washing twice with PBS, cells were observed with an Automatic inverted fluorescence microscope (Leica Microsystems, DMI4000B).

Cell culture and Cell viability assay

For in vitro experiments, Hepatocytes and L929 cells were cultured in a cell incubator at 37 °C under 5% CO_2. The medium was high-glucose Dulbecco's modified Eagle medium (DMEM) with 10% fetal bovine serum (FBS), penicillin/streptomycin (100 $\mu g \cdot mL^{-1}$). H22 cells were also routinely cultured in RPMI-1640 medium with 10% (v/v) FBS and 1% (w/v) penicillin/streptomycin at 37 °C in a humidified atmosphere containing 5% CO_2. In the ascites model, H22 cells were diluted with RPMI-1640 medium to a concentration of 1×10^7 $cells \cdot mL^{-1}$, and then 0.5 mL of cell suspension was injected into the abdominal cavity of ICR mice. The mice were sacrificed after 7 d, and H22 cells were collected from the ascites.

The cell cytotoxicity of as-prepared materials was evaluated with cell counting kit 8 (CCK-8) assay. In brief, 100 μL H22 cells, L929 cells and Hepatocytes were seeded in 96-well plate at a density of 5×10^3 cells/well and incubated in a 5% CO2 incubator at 37 °C for 24 h, replacing the culture medium with fresh RPMI-1640 and DMEM

contained different concentrations of nanomaterials (0, 25, 50, 100μg/mL) and further incubated for 12 h. Then cells in each well were incubated with 10 μL CCK8 solution for another 2 h. Finally, the absorbance at 450 nm was recorded using a microplate reader.

Cell death form confirmation

To study the specific cell death pathway included by our formulation, nanomaterials was used in combination with Fer-1 (Ferroptosis inhibitor, 2 μM), ZVAD-FMK (apoptosis inhibitor, 1μM), 3-MA (autophagy inhibitor, 50 μM) and Necrostatin-1 (necrosis inhibitor, 1μM) to measure the cell viability by the CCK8 assay.

Intracellular ROS and Lipid ROS measurement

The intracellular ROS and lipid ROS generation by nanomaterials was measured by flow cytometry by respectively using a H2DCFDA and C11-BODIPY probe. Briefly, 1.0×10^5 H22 cells were seeded into 12-well plates and allowed to settle 24 h for adherence. Different concentrations of nanomaterials (0, 25,50, 100 μg/mL) were added into wells for 12 h incubation. For detection of cellular ROS, 20 μM H2DCFDA was incubated with H22 cells at 37 °C in dark for 30 min. Lipid ROS was tested by assessing the fluorescence levels upon staining with 5 μM CD11-BODIPY for 30 min at 37 °C in dark. Then, H22 cells were rinsed with PBS three times and analyzed by flow cytometry.

2′,7′-Dichlorodihydro-fluorescein diacetate (DCFH-DA) was used to further detect intracellular ROS [11]. H22 cells were incubated with different nanomaterials (ZIF-8, DOX, ZIF-8@DOX, ZIF-8/SrSe, ZIF-8/SrSe@DOX) for 6 h. After that, a DCFH-DA solution (0.2 μM) was added to cells for 15 min at 37°C in the dark. After washes with PBS, images were captured using a Automatic inverted fluorescence microscope (Leica Microsystems, DMI4000B). The fluorescence was obtained at 495 nm (excitation) and 529 nm (emission).

Intracellular ferrous Ion (Fe^{2+}) assay

To measure intracellular ferrous iron (Fe^{2+}) level, 1×10^5 H22 cells were seeded into 12-well plates. When the cells reached about 70% of the plates, cells were treated with different concentrations of nanomaterials (0, 25,50, 100 μg/mL) for 12 h, and then

incubated with 1μM Far-Red Labile Fe^{2+} dye at 37 °C for 30 min in the dark. After washing with PBS, the intracellular Fe^{2+} levels were analyzed by flow cytometry.

Measurement of glutathione (GSH) and malondialdehyde (MDA) content

H22 cells (2×10^5) in 6-well plate were treated as indicated, and cellular GSH and MDA content was assessed using the GSH assay kit (Beyotime Biotechnology) according to the manufacturer's instructions.

Cell apoptosis

H22 cells (1×10^5 per well) were seeded into 12-well plates and cultured overnight. Subsequently, cells were treated with as-prepared nanomaterials as indicated, and apoptotic cell death was detected by Alexa Fluor® 647-conjugated Annexin V with the DAPI staining assay, according to the manufacturer's protocols. The stained cells were diluted by binding buffer and suspended. FACS was used to test the population of apoptotic cells.

Cancer therapy evaluation *in vitro*

The technology of Annexin V-FITC/DAPI double staining was operated according to the manufacturer's instructions. Briefly, after incubating with ZIF-8/SrSe@DOX for 6 h, H22 cells were collected, washed with PBS, and suspended into 200 μL binding buffer. Then cells were stained in the dark for 15 min by 2.5 μL Annexin V-FITC and 5 μL DAPI. Finally, the stained cells were analyzed by a flow cytometer.

Mitochondrial morphological analysis

For transmission electron microscopy (TEM) analysis, H22 cells were seeded into 6-well plate (2×10^5 per well) and cultured overnight. Then the cells were treated with 50 μg/mL ZIF-8/SrSe@DOX for 12 h. For intracellular structure observation, cells were harvested and fixed in 2.5 % glutaraldehyde for 12 h, and postfixed in 1% osmium tetroxide for 2 h and stained with 0.5% uranyl acetate. Next, each sample was diluted with absolute alcohol and then applied onto copper grids followed by vacuum drying. Images were obtained by using a transmission electron microscope (TEM, a Tecnai 10 microscope).

Western blotting

To demonstrate the expression of ferroptosis-related and apoptosis-related proteins: Transferrin Receptor 1(TFR1), Recombinant Ferroportin (FPN), Recombinant Ferritin, Light Polypeptide (FTL), NF-E2-related factor 2(NRF2), Heme oxygenase-1 (HO-1), Recombinant Glutathione Peroxidase 4 (GPX4), P62 protein (P62), Cytoplasmic linker protein (Keap1), Recombinant Solute Carrier Family 7, Member 11 (SLC7A11), Caspase-3 and Caspase-9. A western blotting assay was performed as described previously [12]. Briefly, H22 cells were seeded in 6-well plate and cultured for 12 h. After incubating with different nanomaterials for 12 h. The cell samples were obtained and lysed in RIPA buffer with protease inhibitors on ice for 30 min. Protein concentrations were detected by BCA protein assay reagent. Proteins were separated on SDS-PAGE and transferred to PVDF membranes. Membranes were blocked with 5% nonfat milk and further incubated with the indicated primary antibodies overnight at 4 °C. Subsequently, membranes were incubated with HRP-conjugated secondary antibody for 1 h at room temperature and scanned with a Tanon 5200 imaging analysis system (Tanon, Shanghai, China). The gray-scale intensity of various ferroptosis-related proteins was assessed by using ImageJ software.

Animal experiments

The animal study was performed according to protocols approved by animal Ethics Committee of Sichuan Agricultural University. For tumor inhibition evaluation, H22 cells tumor-bearing mice were randomly divided into six groups (n = 6). When the tumor volume was about 100 mm^3, the mice were treated with saline; ZIF-8; DOX; ZIF-8@DOX; ZIF-8/SrSe; ZIF-8/SrSe@DOX. The body weight and tumor volume were monitored every 2 days. The tumor volumes were calculated by the following formula: volume (mm^3) = length × $width^2/2$. After ten days, the mice were euthanized, and the tumors were excised for weight and volume assessments. Concurrently, the heart, liver, spleen, lung, and kidney tissues were harvested for subsequent hematoxylin and eosin (H&E) staining.

For in vivo biosafety evaluation, female mice aged 6 weeks were purchased from Chengdu Dashuo experimental animal Co., Ltd. Healthy mice were randomly divided

into 4 groups with 4 mice in each group. These included saline (observed for 7 and 28 days) and ZIF-8/SrSe@DOX (36 mg/kg, observed for 7 or 28 days). Mice were injected with ZIF-8/SrSe@DOX through tail vein. Body weight was measured at the same time every day for 28 days to monitor the physical health of each mouse. The mice were euthanized after the designated time of feeding and observation, and their whole blood samples were collected for standard hematology test and serum biochemical analysis. Meanwhile, the heart, liver, spleen, lung, kidney and other major organs were fixed in 4% paraformaldehyde for standard HE staining.

Hemolysis assay *in vitro*

50 μL of fresh mice blood was added into 1.95 mL of ZIF-8/SrSe NPs saline solution with different concentrations (0, 6.125, 12.5, 25, 50, 100 μg mL^{-1}), respectively. Simultaneously, as a control, 50 μL of fresh blood was added into 1.95 mL of deionized water. After 12 h of incubation, all samples were centrifuged and the absorbance of the supernatant was measured using a UV–vis spectrophotometer (500-600 nm).

Statistical analysis of experimental data

All data were presented as the mean with the standard deviation (Mean±SD). The differences between multiple experimental groups were analyzed by one-way analysis of variance (ANOVA), and the statistical comparison between two groups employed the two-tailed, independent student-t test. The tests were regarded as statistically significant at $*p < 0.05$ (significant), $**p < 0.01$ (moderately significant), and $***p < 0.001$ (highly significant).

Supplementary Figures and Tables.

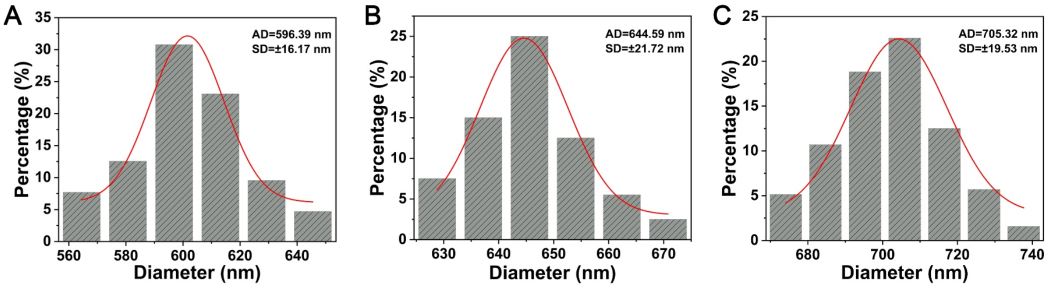

Figure S1. Statistical analysis of the lateral sizes of (A) ZIF-8, (B) ZIF-8/SrSe, (C) ZIF-8/SrSe@DOX determined by SEM.

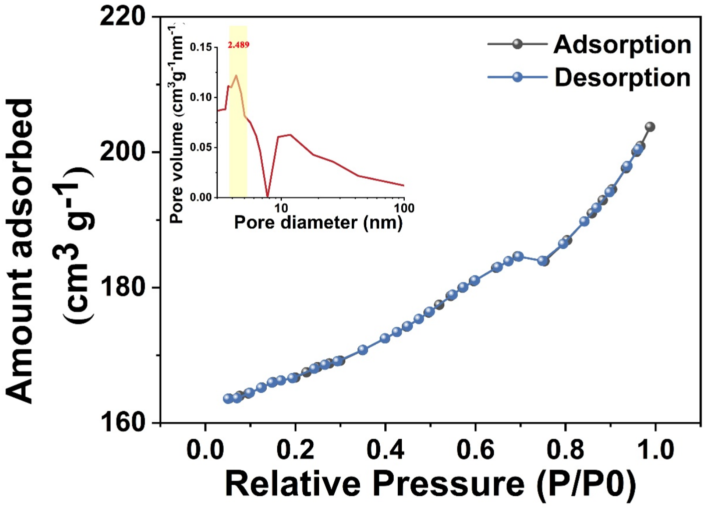

Figure S2. Nitrogen adsorption-desorption isotherm and pore size distribution plot of the ZIF-8/SrSe@DOX NPs.

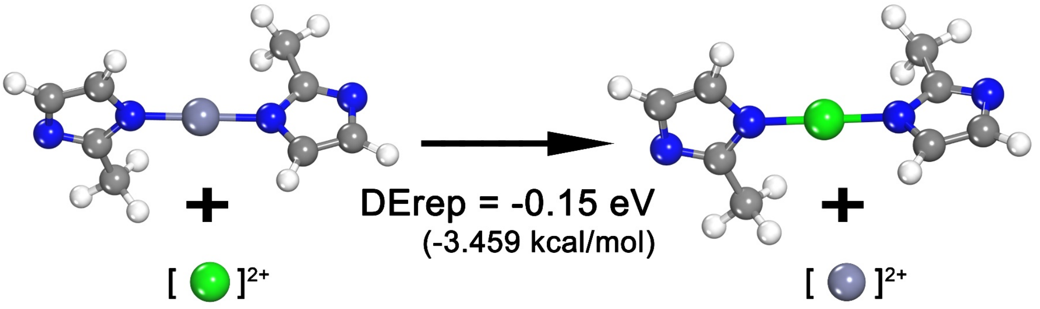

Figure S3. Energy change in the replacement of Zn $(Im)_2$ to Sr $(Im)_2$. (The green, purple, blue, gray and white balls represent the Sr, Zn, N, C and H atoms, respectively.)

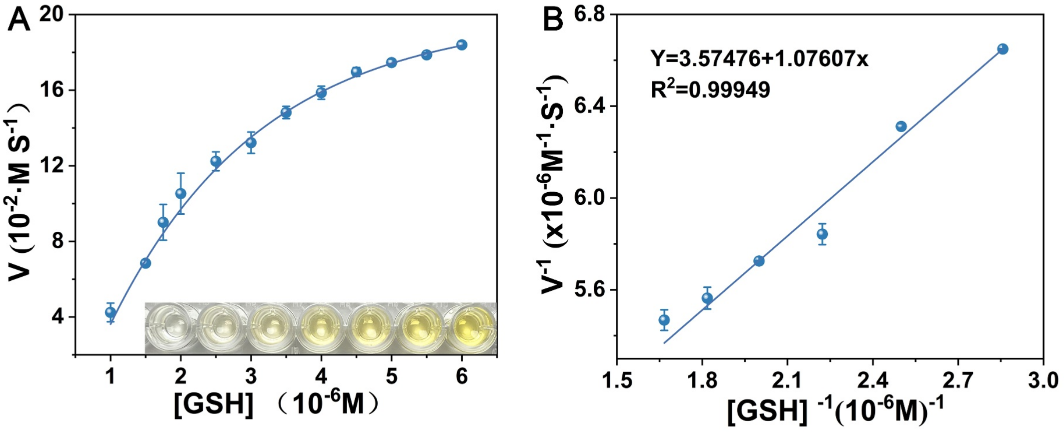

Figure S4. (A) Steady-state kinetic assay and (B) Lineweaver−Burk plot for ZIF-8/SrSe. The inset shows the color change of GSH after ZIF-8/SrSe treatment by DTNB colorimetric assay.

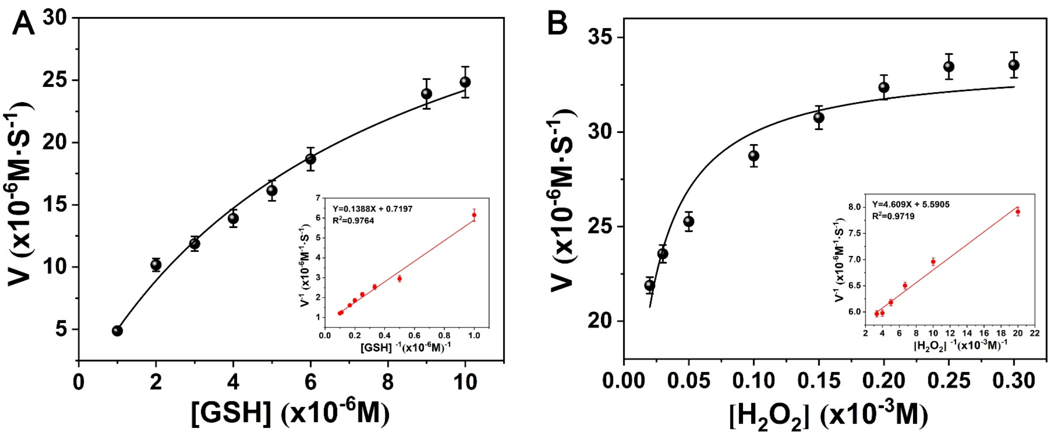

Figure S5. Steady-state kinetic profiles of the GPX-like activity for ZIF-8/SrSe. (A) GSH as substrate and (B) H_2O_2 as substrate.

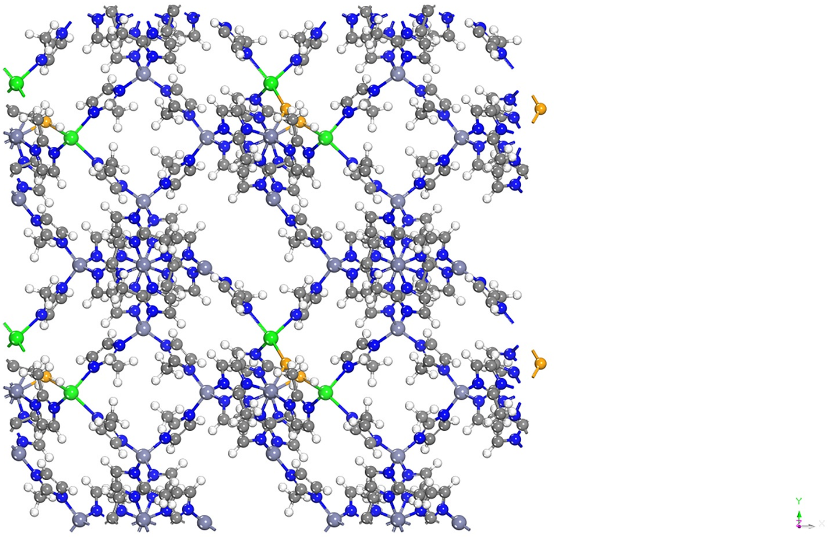

Figure S6. The optimized structures of ZIF-8/SrSe nanozyme structure. (The green, Orange, blue, gray and white balls represent the Sr, Se, N, C and H atoms, respectively.)

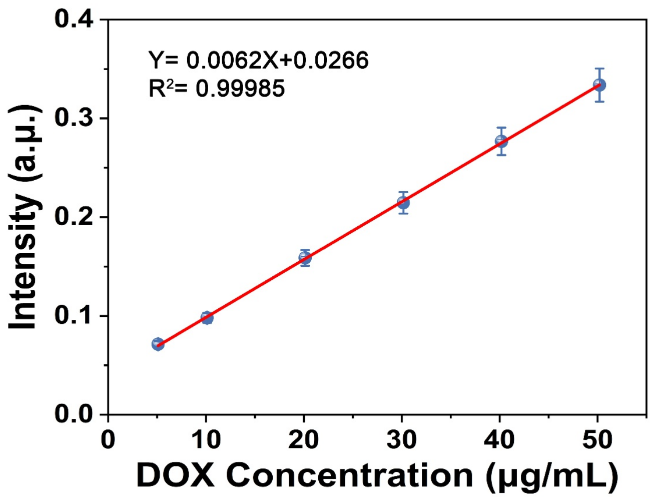

Figure S7. The standard curve of absorption intensity of DOX as a function of concentration. The absorption intensity was measured at 480 nm.

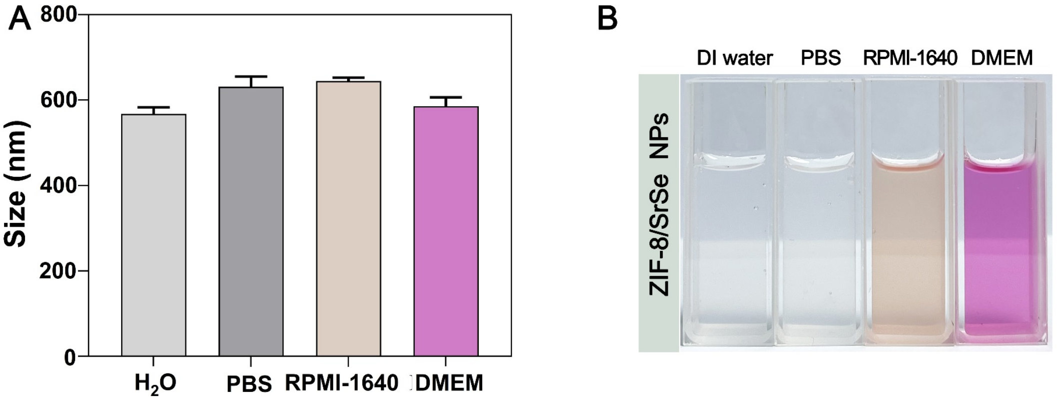

Figure S8. (A) The hydrodynamic sizes and (B) the digital photo of ZIF-8/SrSe nanozyme dispersed in DI water, PBS, RPMI-1640, and DMEM solutions.

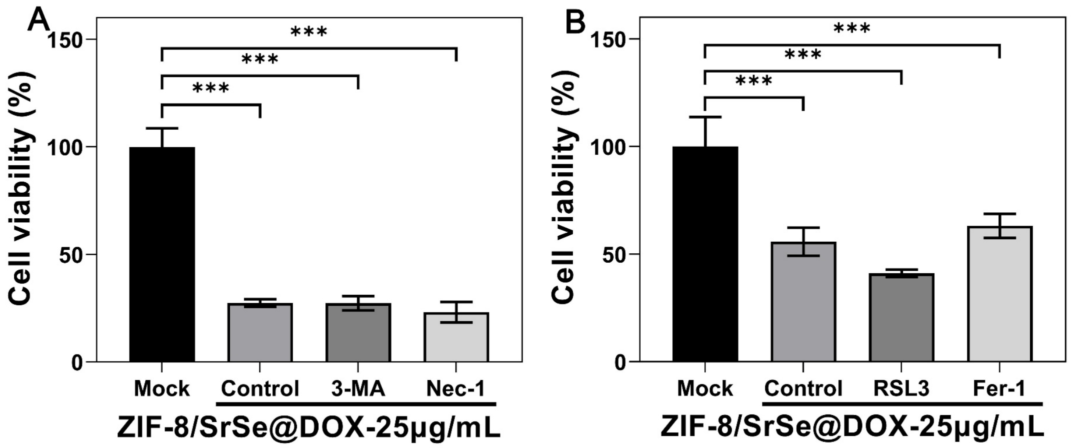

Figure S9. (A) Cell viability was assayed by CCK-8 in ZIF-8/SrSe@DOX-treated H22 cells treated with or without 3-Methyladenine (3-MA, an autophagy inhibitor) and Nec-1 (necroptosis inhibitor) (n=8). (B) Cell viability was assayed by CCK-8 in ZIF-8/SrSe@DOX-treated H22 cells treated with or without RSL3 (ferroptosis activator) and Fer-1 (ferroptosis inhibitor) (n=8). (*P < 0.05, **P < 0.01, ***P < 0.001).

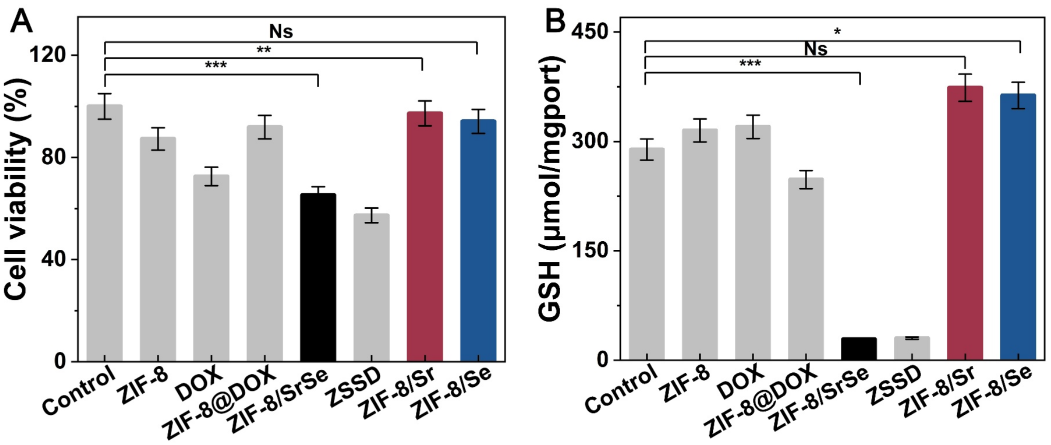

Figure S10. (A) Effect of ZIF-8/Sr, ZIF-8/Se and ZIF-8/SrSe-treated H22 cells viability (n=5). (B) The content of glutathione (GSH) was measured in ZIF-8/Sr, ZIF-8/Se and ZIF-8/SrSe-treated H22 cells (n=5). (*P < 0.05, **P < 0.01, ***P < 0.001).

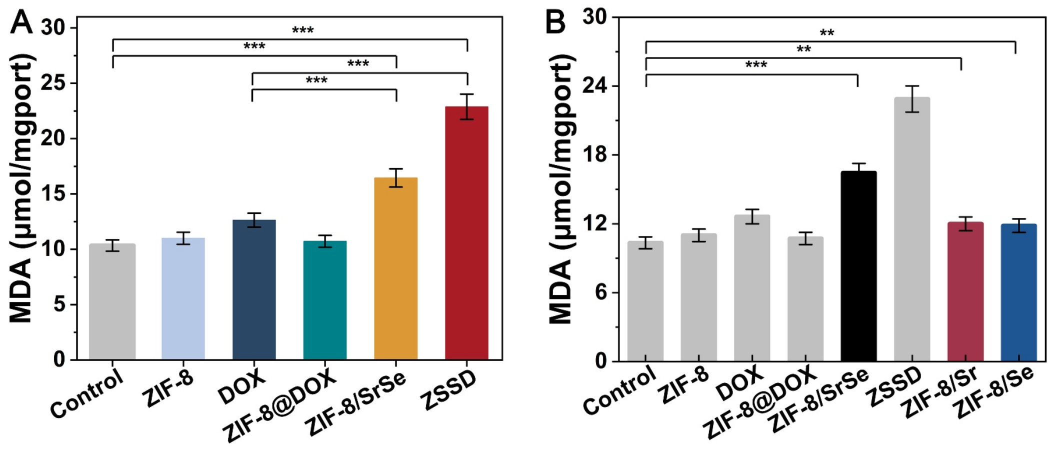

Figure S11. The content of malondialdehyde (MDA) was measured in ZIF-8, DOX, ZIF-8@DOX, ZIF-8/SrSe, ZIF-8/SrSe@DOX, ZIF-8/Sr and ZIF-8/Se -treated H22 cells (n=5). (*P < 0.05, **P < 0.01, ***P < 0.001).

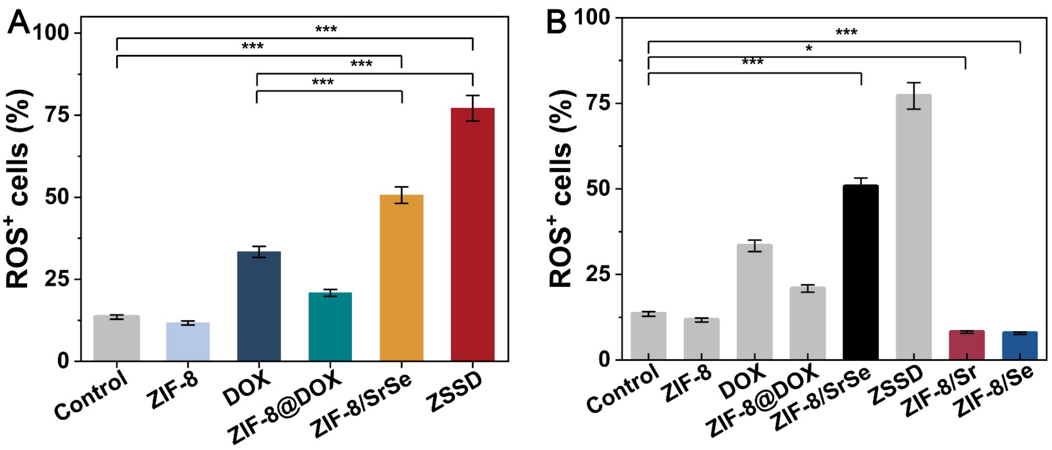

Figure S12. Total cytosolic ROS level assessed overtime by FACS using H2DCFDA (n=5). (*P < 0.05, **P < 0.01, ***P < 0.001).

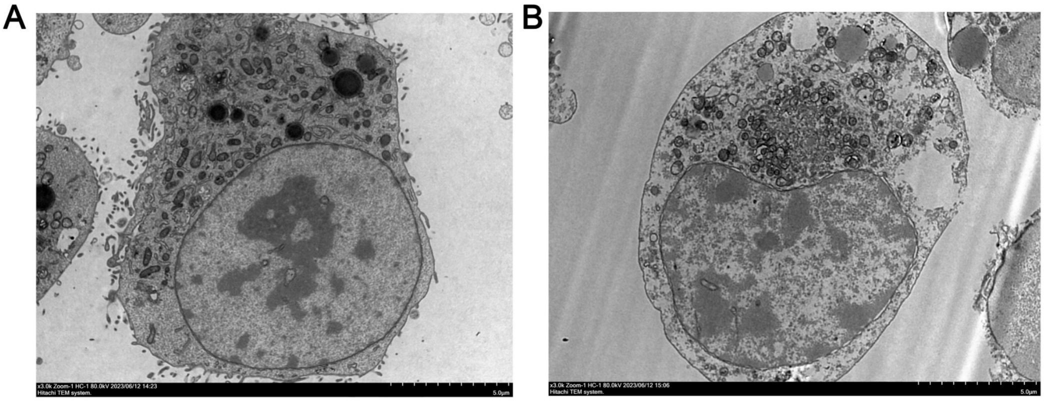

Figure S13. (A) TEM of the morphology of H22 cells treated with PBS and (B) ZIF-8/SrSe@DOX NPs.

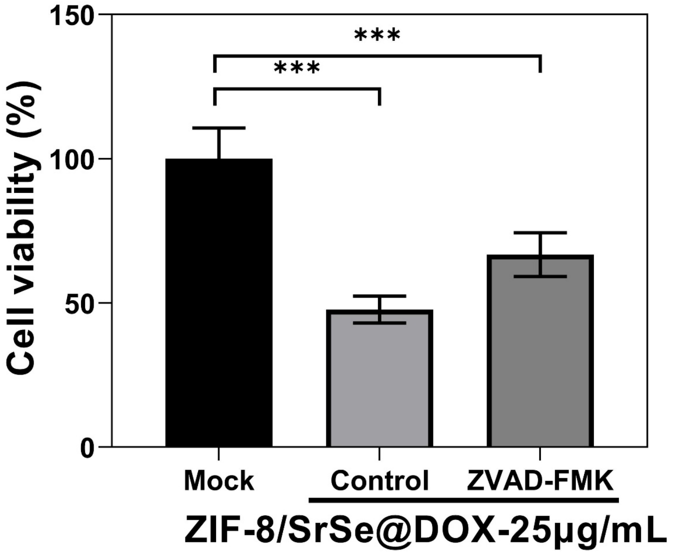

Figure S14. Cell viability was assayed by CCK-8 in ZIF-8/SrSe@DOX-treated H22 cells treated with or without ZVAD-FMK (an apoptosis inhibitor) (n=5). (*P < 0.05, **P < 0.01, ***P < 0.001).

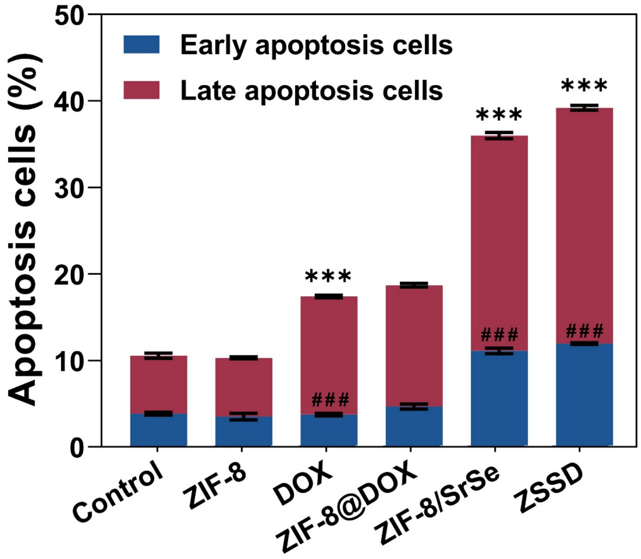

Figure S15. Early and late apoptosis data of H22 cells after different experimental treatments. (#represents early apoptosis cells, * represents late apoptosis cells. #, *P < 0.05, ##, **P < 0.01, ###, ***P < 0.001).

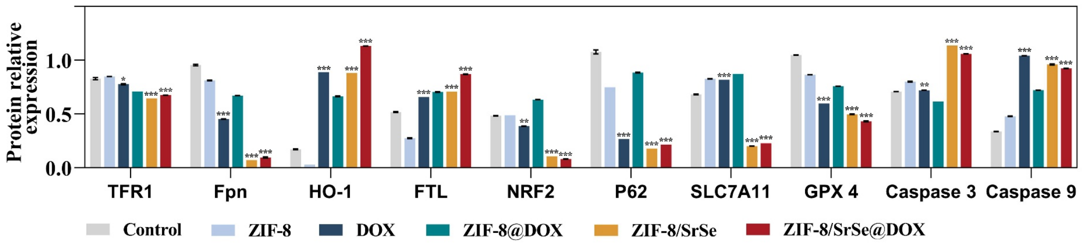

Figure S16. Quantification of related proteins expression level in materials-treated cancer tissue (*P < 0.05, **P < 0.01, ***P < 0.001).

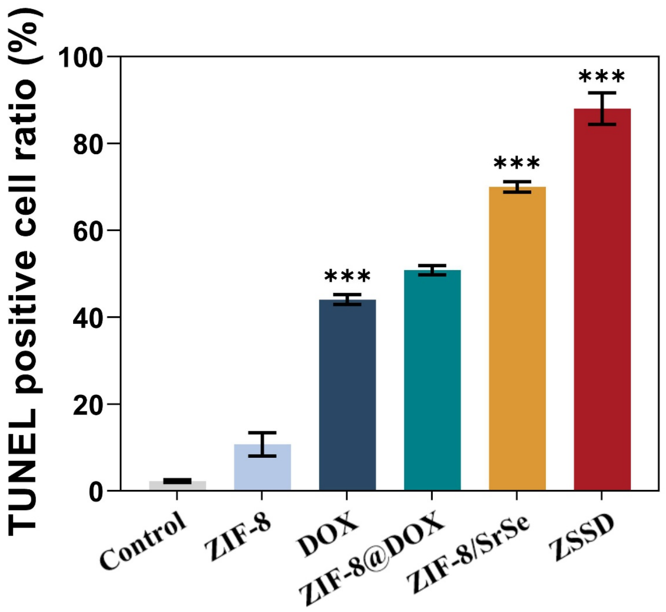

Figure S17. Quantification of TUNEL immunofluorescence staining of cancer tissues treated with different materials (*P < 0.05, **P < 0.01, ***P < 0.001).

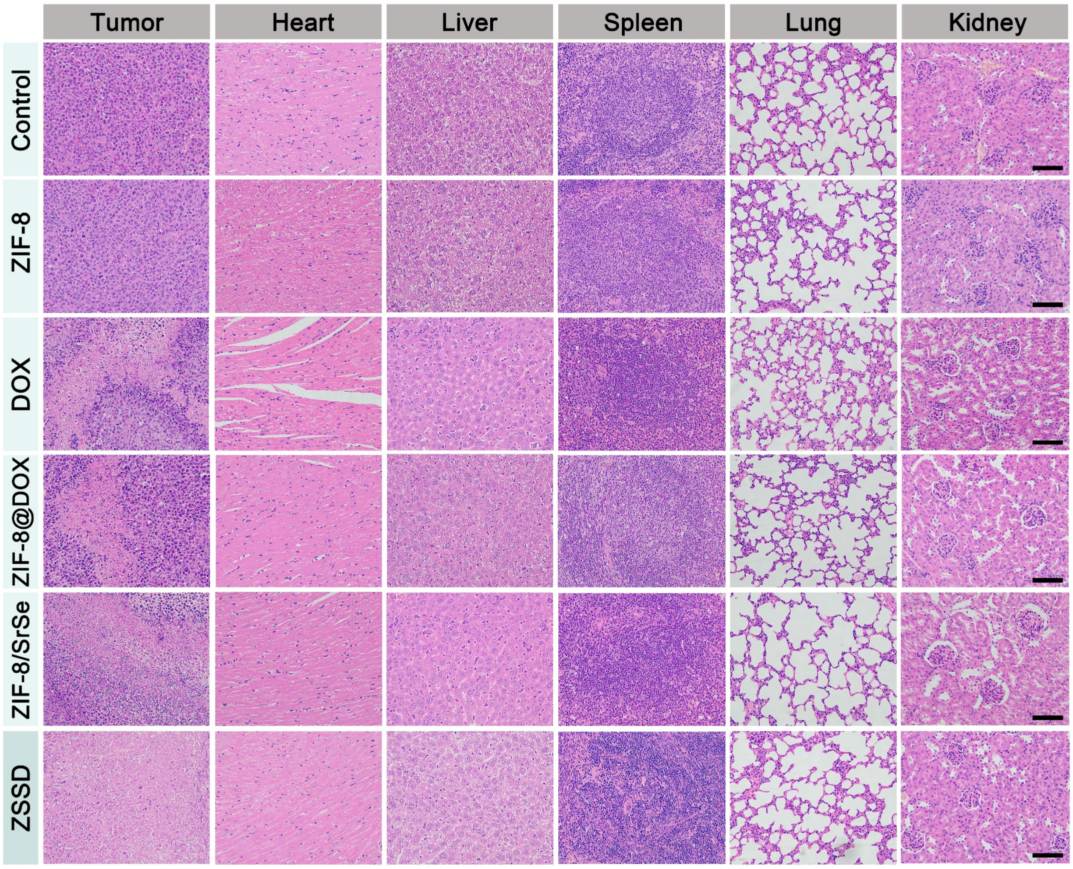

Figure S18. H&E-stained images of tumor and major organs from treated mice. (Scale bar: 100 μm).

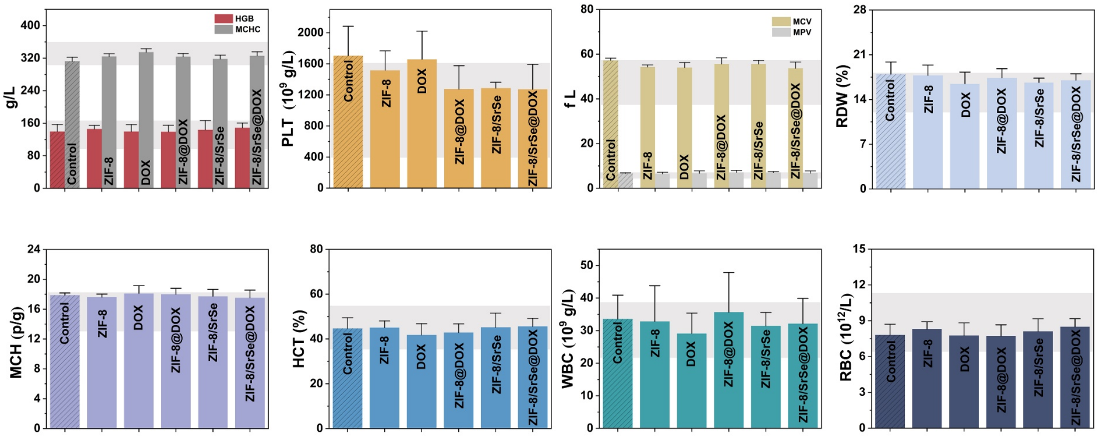

Figure S19. The column chart of primary indicators of blood routine test on day 13 after the mice being intravenously injected with different formulations. Grey hatched area represents the normal reference ranges of hematology data of healthy mice (n=6).

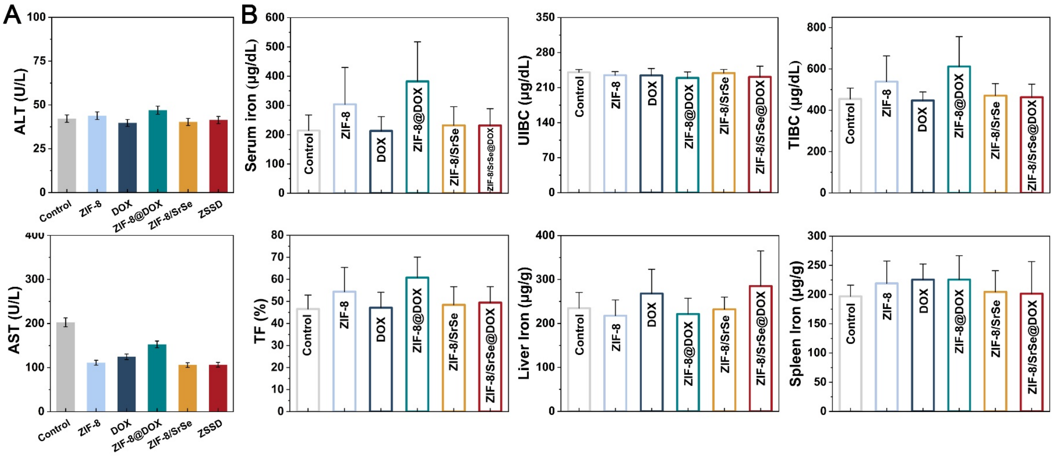

Figure S20. (A) Serum biochemistry of liver function markers: alanine aminotransferase (ALT) and aspartate aminotransferase (AST). (B) Serum iron, UIBC, TIBC, TF and iron content of important tissues (liver and spleen) at the 13 days after tumor treatment (n=6).

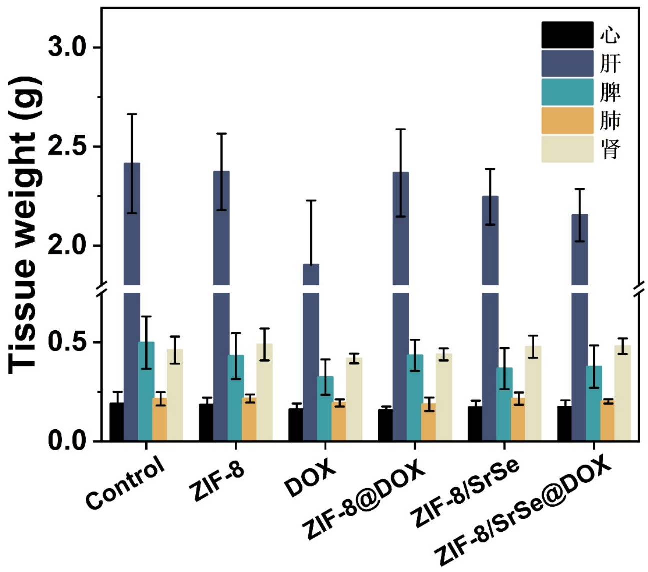

Figure S21. Major organ weights in tumor treatment mice after treated with different formulations. (n=6).

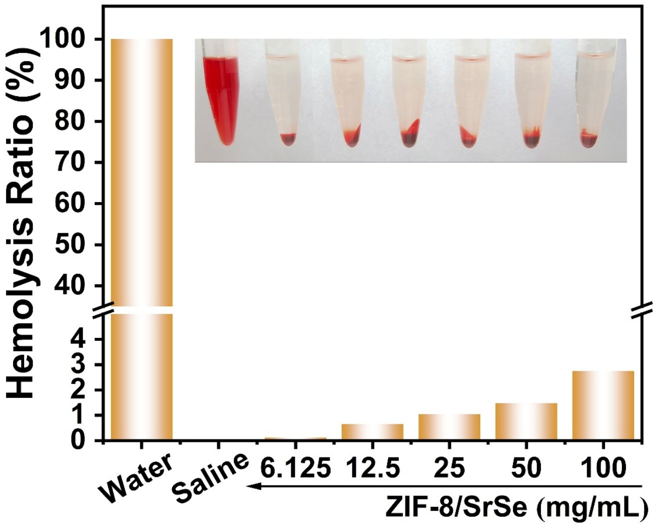

Figure S22. Hemolytic rate of erythrocytes after incubation with ZIF-8/SrSe@DOX at various concentrations at 37°C for 6h. DI water served as the positive control (100% of hemolysis rate). Inset: photograph of the centrifuge tubes containing various samples. Data are shown as mean ± S.D. (n = 3).

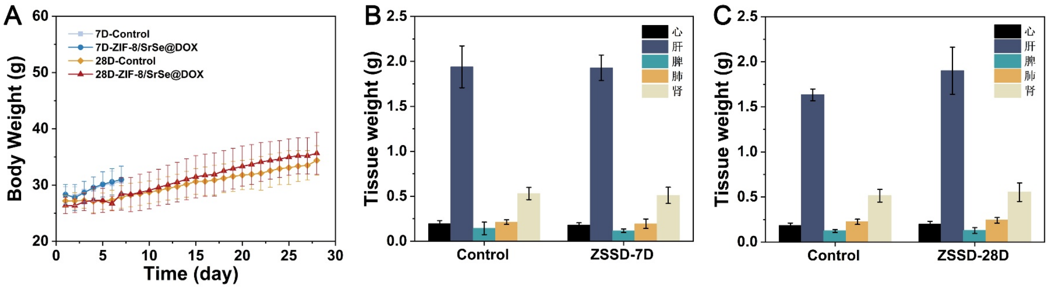

Figure S23. (A) Body weight changes in healthy mice 7 and 28 days after the treatment with ZIF-8/SrSe@DOX. Major organ weights in healthy mice 7 (B) and 28 (C) days after the treatment with ZIF-8/SrSe@DOX (n=6).

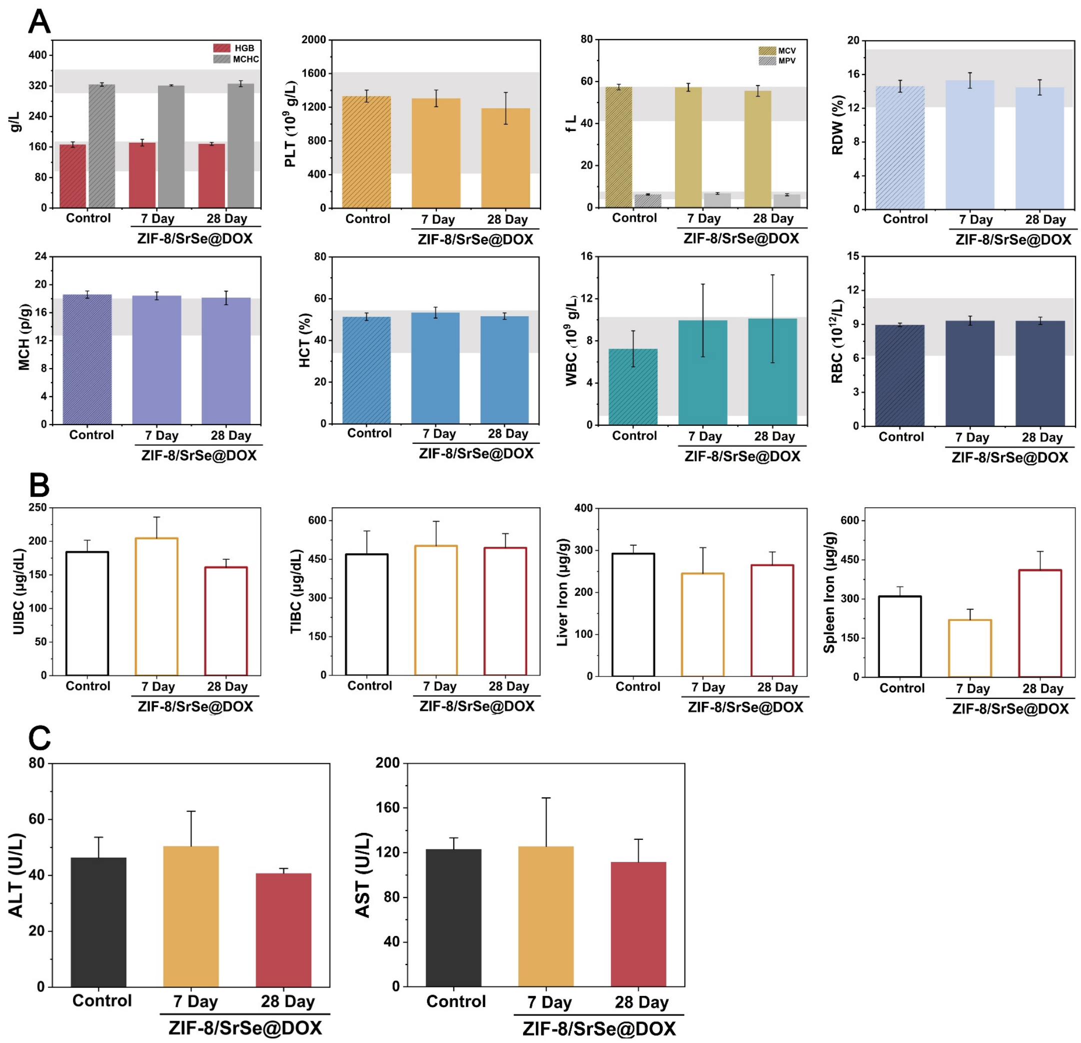

Figure S24. (A) column chart of primary indicators of blood routine test on day 7 and day 28 after the mice being intravenously injected with ZIF-8/SrSe@DOX. Grey hatched area represents the normal reference ranges of hematology data of healthy mice. (B) UIBC, TIBC and iron content of important tissues (liver and spleen) on day 7 and day 28 after the mice being intravenously injected with ZIF-8/SrSe@DOX. (C) Serum biochemistry of liver function markers: alanine aminotransferase (ALT) and aspartate aminotransferase (AST).

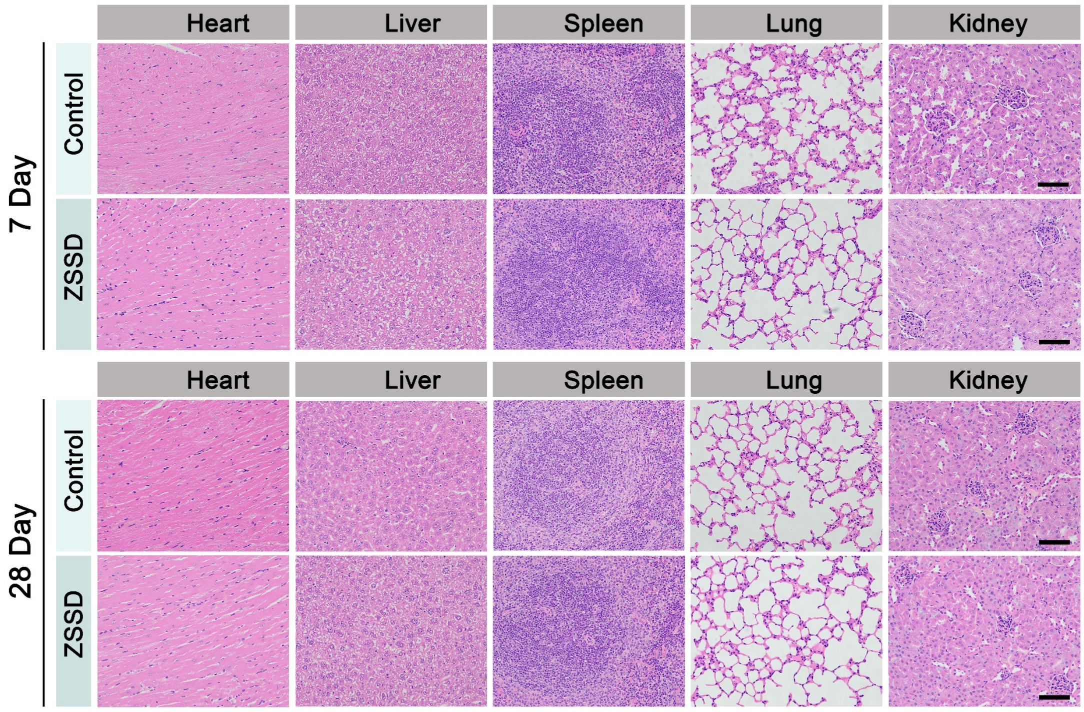

Figure S25. H&E-stained images of major organs from healthy mice 7 and 28 days after the treatment with ZIF-8/SrSe@DOX. (Scale bar: 100 μm).

2. Supplementary tables.

Table S1. The physical properties of as-prepared samples.

Samples	S_{BET} (m^2 g^{-1})	V_{pore} (cm^3 g^{-1})	D_{pore} (nm)
ZIF-8/SrSe	707.7	0.5529	3.125
ZIF-8/SrSe@DOX	506.5	0.3152	2.489

Table S2. DFT calculation study of the energy change of Zn(Im)2 replacement to Sr(Im)2.

Configuration	ENERGY (a.u.)	Delta E (eV)
Zn^{2+}	-226.1176273	
Sr^{2+}	-30.0689705	
Zn-2Im	-757.0345793	
Sr-2Im	-560.9914968	-0.151682763

Table S3. Blood biochemistry and hematology data of female Kunming mice at 7 days 、 28 days and during tumor treatment Period .(n=4, mean ± SD).

Project Name	Treatment Group	7-day Administration Group	28-day Administration Group	Standard indicators	units
	Mean ± SD	Mean ± SD	Mean ± SD		
MPV	6.82±0.95	6.78±0.43	6.14±0.48	4.0-6.2	fL
PLT	1272.2±321.87	1306±99.49	1187.6±188.75	400-1600	10^9/L
RDW	17.02±1.00	15.28±0.92	14.46±0.90	12-39	%
MCHC	326±9.72	321.6±1.51	326±7.87	300-360	g/L
MCH	17.52±1.03	18.4±0.56	18.1±0.97	13.0-18.0	pg
MCV	53.72±2.77	57.24±1.87	55.52±2.54	41.0-55.0	fL
HCT	45.6±3.58	53.36±2.60	51.66±1.54	35.0-55.0	%
HGB	148.8±11.71	171.4±8.79	168.2±3.96	110-165	g/L
WBC	32.168±7.72	9.94±3.45	10.1±4.16	0.80-35	10^9/L
RBC	8.50±0.67	9.324±0.40	9.31±0.32	6.50-11.50	10^{12}/L

References:

1. Andrae D, H?U?Ermann U, Dolg M, Stoll H, Preu H. Energy-adjustedab initio pseudopotentials for the second and third row transition elements. Theor. Chim. Acta. 1990; 77: 123-41.
2. Stephens PJ, Devlin FJ, Chabalowski CF, Frisch MJ. Ab initio calculation of vibrational absorption and circular dichroism spectra using density functional force fields. J. Phys. Chem. 1994; 98: 11623-7.
3. Weigend F, Ahlrichs R. Balanced basis sets of split valence, triple zeta valence and quadruple zeta valence quality for H to Rn: Design and assessment of accuracy. PCCP. 2005; 7: 3297-305.
4. Vandevondele J, Krack M, Mohamed F, Parrinello M, Chassaing T, Hutter J. QUICKSTEP: Fast and accurate density functional calculations using a mixed Gaussian and plane waves approach. Comput. Phys. Commun. 2005; 167: 103-28.
5. Hartwigsen C, Goedecker S, Hutter J. Relativistic separable dual-space Gaussian Pseudopotentials from H to Rn. Phys. Rev. B 1998; 58: 3641-62.
6. Vandevondele J, Hutter J. Gaussian basis sets for accurate calculations on molecular systems in gas and condensed phases. J. Chem. Phys. 2007; 127: 4365-477.
7. John, P., Perdew, Kieron, Burke, Matthias, et al. Generalized Gradient Approximation Made Simple. Phys. Rev. Lett.. 1996.
8. Grimme S, Antony J, Ehrlich S, Krieg H. A consistent and accurate ab initio parametrization of density functional dispersion correction (DFT-D) for the 94 elements H-Pu. J. Chem. Phys. 2010; 132: 154104.
9. Forman HJ, Zhang H, Rinna A. Glutathione: Overview of its protective roles, measurement, and biosynthesis. Mol. Aspects Med. 2008; 30: 1-12.
10. Zhu X, PengGe, YuWu, RuimeiXue, TingSheng, YingyingAi, ShirongTang, KaijieWen, Yangping. MoS2/MWCNTs porous nanohybrid network with oxidase-like characteristic as electrochemical nanozyme sensor coupled with machine learning for intelligent analysis of carbendazim. J. Electroanal. Chem.: 2020; 862.
11. Armstrong, Donald. [Methods in Molecular Biology] Advanced Protocols in Oxidative Stress II Volume 594 || Identification of ROS Using Oxidized DCFDA and Flow-Cytometry. 2010; 10.1007/978-1-60761-411-1: 57-72.
12. Li C, Zhang Y, Liu J, Kang R, Klionsky DJ, Tang D. Mitochondrial DNA stress triggers autophagy-dependent ferroptotic death. Autophagy. 2021; 17: 948-60.
